# Self-adjuvanting nanovaccines boost lung-resident CD4^+^ T cell immune responses in BCG-primed mice

**DOI:** 10.1038/s41541-022-00466-0

**Published:** 2022-04-26

**Authors:** Megan A. Files, Kubra F. Naqvi, Tais B. Saito, Tara M. Clover, Jai S. Rudra, Janice J. Endsley

**Affiliations:** 1 Department of Microbiology and Immunology, University of Texas Medical Branch, Galveston, TX 77555 USA; 2 Institute of Translational Science, University of Texas Medical Branch, Galveston, TX 77555 USA; 3 Department of Internal Medicine, University of Texas Southwestern Medical Center, Dallas, TX 75390 USA; 4 Department of Pathology, University of Texas Medical Branch, Galveston, TX 77555 USA; 5 Comprehensive Industrial Hygiene Laboratory (CIHL), Navy Environmental and Preventive Medicine Unit TWO (NEPMU-2), Department of the Navy, Norfolk, VA 23551 USA; 6 Department of Biomedical Engineering, McKelvey School of Engineering, Washington University in St. Louis, St. Louis, MO 63130 USA; 7 Present Address: Laboratory of Bacteriology, Division of Intramural Research, National Institute of Allergy and Infectious Diseases, National Institutes of Health, Hamilton, MT 59840 USA

**Keywords:** Cellular immunity, Peptide vaccines

## Abstract

Heterologous vaccine regimens could extend waning protection in the global population immunized with *Mycobacterium bovis* Bacille Calmette-Guerin (BCG). We demonstrate that pulmonary delivery of peptide nanofibers (PNFs) bearing an Ag85B CD4^+^ T cell epitope increased the frequency of antigen-specific T cells in BCG-primed mice, including heterogenous populations with tissue resident memory (Trm) and effector memory (Tem) phenotype, and functional cytokine recall. Adoptive transfer of dendritic cells pulsed with Ag85B-bearing PNFs further expanded the frequency and functional repertoire of memory CD4^+^ T cells. Transcriptomic analysis suggested that the adjuvanticity of peptide nanofibers is, in part, due to the release of damage-associated molecular patterns. A single boost with monovalent Ag85B PNF in BCG-primed mice did not reduce lung bacterial burden compared to BCG alone following aerosol *Mtb* challenge. These findings support the need for novel BCG booster strategies that activate pools of Trm cells with potentially diverse localization, trafficking, and immune function.

## Introduction

Tuberculosis (TB) remains one of the leading infectious causes of death despite a century of routine BCG vaccination. A quarter of the global population is estimated to be latently infected with *Mycobacterium tuberculosis* (*Mtb*) and serve as a large human reservoir^1^. In 2019, there were approximately 10 million new cases of tuberculosis (TB), and 1.4 million deaths^2^. *Mtb* remains an eminent threat to global health due to the high burden of TB, particularly in immunocompromised individuals, and especially because of the development and spread of drug-resistant strains. Therefore, subunit vaccine strategies that are safe across all populations and extend BCG-primed immunity are urgently needed.

The efficacy of BCG as a vaccine is highly variable with regard to long term protection, although it is fairly effective at preventing childhood forms of TB. Protection begins to wane in adolescence, leaving individuals more susceptible to *Mtb* infection^3^. Use of a homologous BCG booster has been shown to expand humoral responses^4^, but does not provide any further cell-mediated protection^5,6^. Heterologous vaccination strategies utilizing *Mtb*-specific antigens have demonstrated superior T cell responses with protective functions^7,8^. To this end, there are currently a variety of vaccine candidates in the clinical development pipeline, ranging from subunit vaccines to vector-based, live-attenuated, and whole-cell-derived vaccines designed to either replace BCG or boost BCG-induced protection^2^.

A large obstacle to TB vaccine design has been the lack of defined correlates of protection. However, numerous studies have unequivocally demonstrated that T lymphocytes are a critical component to a protective immune response against *Mtb* infection. More specifically, a subset of memory T cells identified as tissue-resident memory T cells^9–12^ (Trm) have protective effects upon adoptive transfer into TCR-deficient *Mtb*-infected animals^13^. These cells are characterized by surface markers, most notably CD44^hi^CD62L^lo^CD69^+^ CCR7^−^, as well as tissue-homing markers, and reside in the lung parenchyma. Importantly, Trms were associated with protection in a study demonstrating protective efficacy of intravenous BCG in non-human primate models of *Mtb* infection^14^.

In the last decade, subunit vaccine design based on natural and synthetic materials has gained considerable attention due to the rich chemistry available for modifying materials to modulate the immune system^15–17^. Notably, *Mtb* vaccines based on polymeric micro and nanoparticles^18,19^, liposomes^20^, and virus-like particles (VLPs)^21^ have shown considerable success in preclinical models of TB^22–24^. Our lab studies peptide nanofibers (PNFs) as self-adjuvanting vaccine delivery vehicles and previous studies have demonstrated that the PNF, KFE8, promotes antigen presentation through autophagic and proteasome-dependent mechanisms^25^ and can induce antigen-specific T cell and B cell responses^26^. In the current work, we test the hypothesis that the KFE8 bearing CD4^+^ T cell epitopes could be used to generate Trms directed toward an *Mtb* antigen of choice in the setting of previous BCG vaccination. Moreover, that pulmonary delivery of the KFE8-Ag85B booster would drive proliferation and retention of these *Mtb*-specific CD4^+^ T cells as tissue-resident cells in the lung parenchyma. A fusion peptide, KFE8-Ag85B, composed of the self-assembling peptide KFE8 (FKFEFKFE) conjugated to Ag85B_240–254_ was generated as a PNF subunit vaccine. BCG-primed C57BL/6 mice were subsequently boosted with KFE8-Ag85B in PBS or KFE8-Ag85B-pulsed DCs through an intratracheal (i.t.) route.

Our results demonstrate that a pulmonary KFE8-Ag85B boost generates robust expansion of memory CD4^+^ T cell pools in lungs of mice with previous BCG exposure, an effect further augmented by the administration of KFE8-Ag85B-pulsed DCs. Phenotypic profiling and i.v. staining demonstrated the generation of Trms in the lung parenchyma, as well as non-resident Teff and Tem cells. Post-boost, memory cells mounted a robust cytokine response to cognate antigen including, Th1, Th2, and Th17 profiles. Consistent with other reports, however, expansion of Ag85B-specific CD4^+^ T cells was not sufficient to significantly increase protective immunity compared to BCG after pulmonary *Mtb* challenge. Transcriptional analysis revealed damage-associated molecular pattern (DAMP) and other immune activation signatures as candidate mechanisms for how KFE8 may activate effector and/or memory responses to cognate antigen. Taken together, our results demonstrate an important proof of concept that KFE8-based subunit TB vaccines can effectively increase pulmonary CD4^+^ Trms and other effector/memory populations that recognize a targeted epitope and program multifunctional cytokine recall. These results set the stage to advance the development of single, or multi-valent, PNF subunit TB vaccines that would expand T cells recognizing subdominant or other protective epitopes as part of a heterologous prime/boost strategy.

## Results

### Boosting with KFE8 subunit vaccine expands antigen-specific CD4^+^ T cell populations

**Fig. 1:**
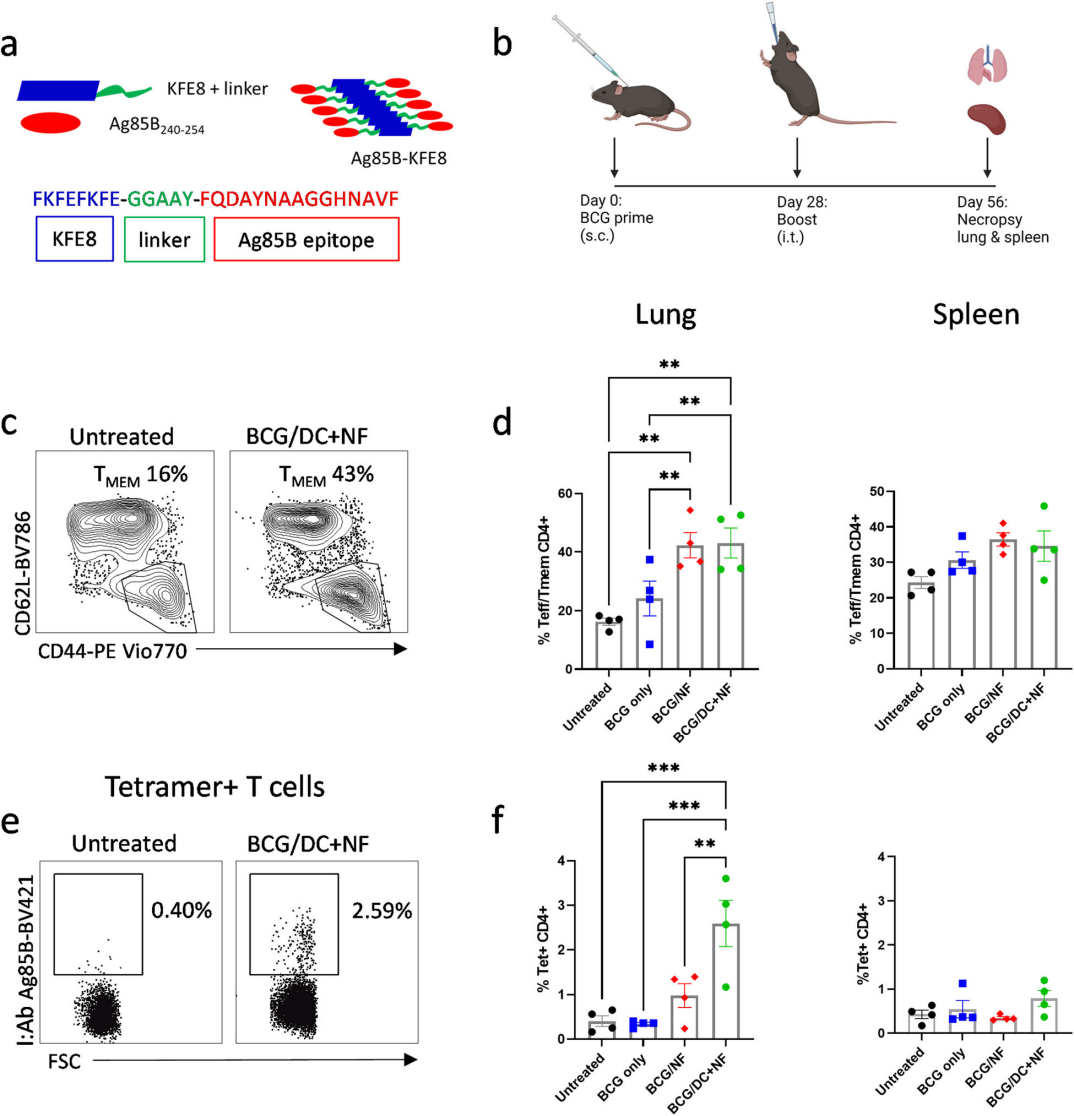
Boost with KFE8-Ag85B vaccine expands antigen-specific CD4^+^ T cell populations in C57BL/6 mice. **a** KFE8 peptide nanofibers are conjugated to Ag85B_240–254_, a CD4^+^ T cell epitope, through a cleavable linker. **b** Mice were primed with a s.c. dose of 100 μL of 5.5 x 10^5^ CFU BCG and then given an i.t. boost of 100 μM KFE8-Ag85B or 1 × 10^6^ KFE8-Ag85B-pulsed DCs 4 weeks later. At 4 weeks post-boost, lung and spleen were harvested and the T cell populations in disrupted tissue were analyzed by flow cytometry (*n* = 4). **c** T cells with a memory or effector phenotype were identified by gating on CD44^hi^ and CD62L^lo^ following selection of CD3^+^ CD4^+^ T cells **d** and were quantified in the lung and spleen. **e** Tetramer positive cells were selected after gating for CD3^+^ and CD4^+^ T cells. **f** The frequency of Ag85B-specific CD4^+^ T cells is represented as a percentage of total CD4^+^ T cells in lung and spleen. Data shown as mean ± SE. Comparisons were made using a one-way ANOVA followed by a Benjamini Krieger Yekutieli post hoc test for multiple comparisons to determine differences due to treatment and among treatment groups. *P*-values < 0.05 were considered statistically significant. *p < 0.05, **p < 0.01, ***p < 0.001, ****p < 0.0001.

The PNFs were constructed with KFE8, a self-associating peptide 8 amino acids in length conjugated to Ag85B_240–254_ through a cleavable linker, GGAAY (Fig. 1a). In aqueous buffers, KFE8 assembles into nanofibers with cross-β structure and Ag85B is displayed on the surface in a multivalent fashion^27^. We previously demonstrated that PNFs bearing *Mtb* antigens do not measurably improve protection in a murine model of TB when used alone as a subunit vaccine^26^. To determine if KFE8-Ag85B can boost memory T cells in BCG vaccinated animals, mice were primed subcutaneously (s.c.) with BCG Pasteur followed by an intratracheal (i.t.) boost with either KFE8-Ag85B or KFE8-Ag85B pulsed DCs in PBS four weeks later (Fig. 1b). The lungs and spleens were harvested four weeks post-boost and the percentage of viable CD4^+^ T cells with activation or memory phenotypes was identified through multi-color flow cytometry analysis of surface markers CD3, CD4, CD44, CD62L, and CCR7 (Supplemental Fig. 1, Fig. 1c, d). In lung and spleen of all groups receiving a booster dose, we observed significant increases in cells with an effector (Teff) or memory (Tmem) (CD3^+^CD4^+^CD62L^lo^CCR7^-^CD44^hi^) phenotype. The percentage of Teff/Tmem cells was also significantly higher for both boosted groups in the lung compared with untreated animals, and represented greater than 40% of the total CD4^+^ T cell population compared to the untreated or BCG vaccinated only groups, with approximately 16% and 24% Teff/Tem respectively (Fig. 1d).

We next analyzed the tetramer^+^ CD4^+^ T cell populations (Fig. 1e) in the lung and spleen and less than 1% of CD4^+^ T cells were found to be Ag85B-specific in the lungs of BCG-primed mice in the absence of a KFE8-Ag85B boost. The lungs of BCG-primed mice boosted with KFE8-Ag85B or DCs pulsed with KFE8-Ag85B showed significantly increased frequencies of tetramer^+^ cells compared with untreated animals (Fig. 1f). Conversely, splenic tetramer^+^ CD4^+^ T cells remained less than 1% of the total CD4^+^ T cell population in all vaccine groups (Fig. 1f). To control for antigen-independent effects of DC treatment, we performed a boost of non-treated DCs in a group of BCG-primed mice (*n* = 4). There was no change in tetramer positivity in the lung (Supplemental Fig. 2a) or spleen (Supplemental Fig. 2b) of this group compared with untreated controls. Similar to the untreated or BCG vaccinated controls, boosting with KFE8-Ag85B or DCs pulsed with KFE8-Ag85B significantly increased the frequency of tetramer positive cells in the lung compared to boosting with non-treated DC (Supplemental Fig. 2a). Together, these data indicate that a pulmonary boost with KFE8-Ag85B in BCG-primed mice increases the pool of activated and antigen-specific CD4^+^ T cells. This outcome was enhanced in the lung by utilizing DCs to deliver KFE8-Ag85B, indicating an optimized antigen presentation effect.

### KFE8-Ag85B-pulsed DCs increase frequency of total and parenchymal CD4^+^ memory T cells in the lung

**Fig. 2:**
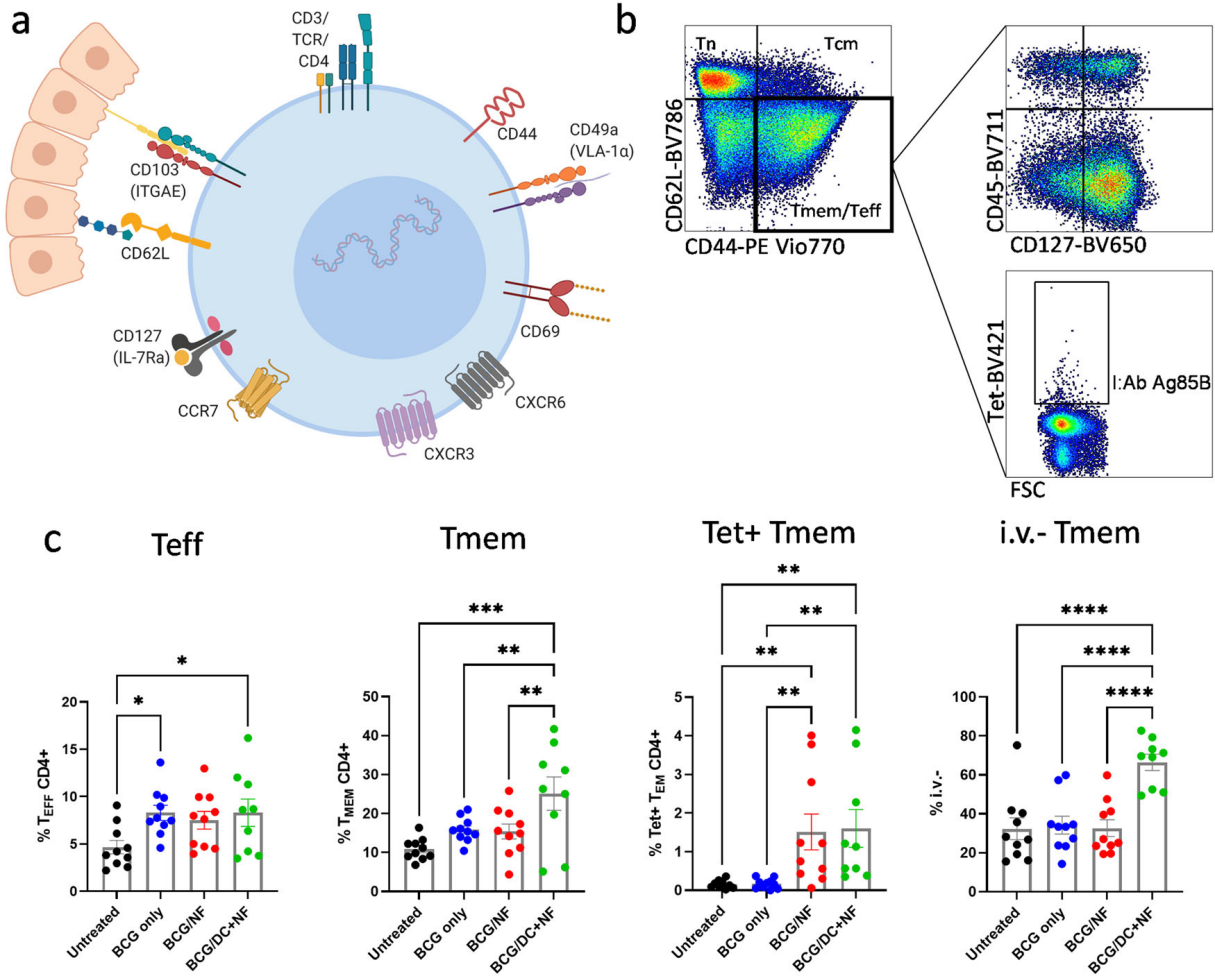
KFE8-Ag85B-pulsed DC delivery increases frequency of total and parenchymal CD4^+^ memory T cells in lungs of BCG-primed mice. **a** Surface markers for tissue resident T cells were selected and used to further characterize KFE8-Ag85B-generated T cells as demonstrated in a graphical representation of lung-resident memory T cells produced in BioRender. **b** Vaccination studies were repeated and the data shown is from lungs of C57BL/6 mice (*n* = 9–10) harvested 4 weeks post boost. CD3^+^ CD4^+^ CCR7^-^ cells were gated on CD44^hi^ and CD62L^lo^, which represent total Teff and Tmem cells. Tmem cells were differentiated from Teff cells by the positive expression of CD127, such that Tmem cells are CD44^hi^ CD62L^lo^ CD127^+^ and Teff cells are CD44^hi^ CD62L^lo^ CD127^−^. Cells were additionally gated on CD45 to determine how cells were distributed between the vasculature (i.v.^+^) and the lung parenchyma (i.v.^-^). Tetramer^+^ cells within the Tmem population were also identified. **c** Frequencies of Teff, Tmem, Tet^+^ Tmem and i.v.^-^ Tmem were determined from CD44^hi^ CD62L^lo^ T cells and expressed as a percentage of the total CD4^+^ T cell population. Data shown as mean ± SE. Significance was determined by one-way ANOVA followed by a Benjamini Krieger Yekutieli post hoc test for multiple comparisons to determine differences due to treatment and among treatment groups. *p < 0.05, **p < 0.01, ***p < 0.001, ****p < 0.0001.

Follow up studies were performed using an expanded profile of selective markers to comprehensively assess memory subpopulations (CD127, CD44, and CD62L) and tissue resident phenotype (CD69, CD49, CD103, CXCR6, and CXCR3) generated by KFE8-Ag85B in a prime/boost regimen with BCG (Fig. 2a). In order to compartmentalize the parenchymal and circulating memory CD4^+^ T cells, mice were also injected i.v. with anti-CD45 antibody just prior to necropsy using a described method^28^. The viable CD3^+^CD4^+^ CCR7^−^T cells were categorized as either Teff (CD127^−^ CD44^hi^ CD62L^lo^) or Tmem (CD127^+^ CD44^hi^ CD62L^lo^) (Fig. 2b). Significant increases in the percentage of Teff CD4^+^ T cells (Fig. 2c) were observed in lungs of mice that were BCG vaccinated and those that were boosted with KFE8-Ag85B-pulsed DCs compared with untreated mice. In contrast, a significant increase in the percentage of Tmem cells was only observed in the KFE8-Ag85B pulsed DC group compared with all other groups (Fig. 2c). Tetramer^+^ CD4^+^ Tmem cells were increased in the lungs of both boosted groups compared with untreated and BCG only controls (Fig. 2c). Analysis of vascular and non-vascular compartments demonstrated that the parenchymal (CD45-, or i.v.-) CD4^+^ Tmem cells were only significantly increased in the lungs of the KFE8-Ag85B-pulsed DC group compared with all groups (Fig. 2c).

### Tissue resident phenotype of CD4^+^ memory T cells in the lung parenchyma is augmented by KFE8-Ag85B boost

**Fig. 3:**
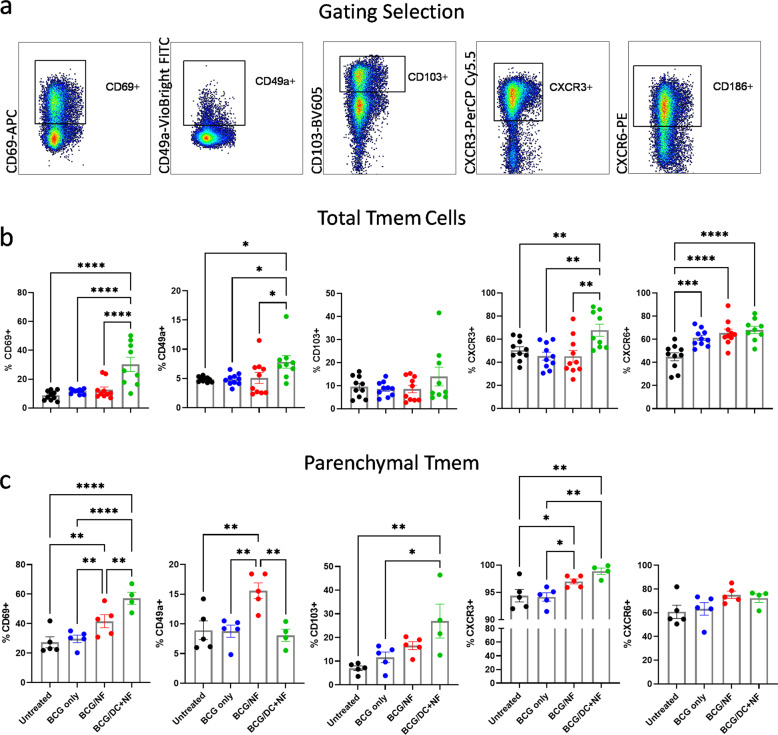
Tissue resident phenotype of CD4^+^ memory cells in the lung parenchyma of BCG-primed mice is augmented by KFE8-Ag85B boost. **a** Memory populations from mouse lungs (*n* = 9–10) were analyzed for common memory markers and tissue localization including CD69, CD49a, CD103, CXCR3 and CXCR6. **b** Frequencies of surface markers are represented as a percentage of the total CD4^+^ Tmem population. **c** Frequencies of surface markers within the parenchymal (i.v.^-^) Tmem population of mouse lung (*n* = 4–5) are also shown. Data shown as mean ± SE. Significance was determined by one-way ANOVA followed by a Benjamini Krieger Yekutieli post hoc test for multiple comparisons to determine differences due to treatment and among treatment groups. *p < 0.05, **p < 0.01, ***p < 0.001, ****p < 0.0001. Unless otherwise noted, comparisons are n.s.

We next characterized the total and parenchymal Tmem cells based on their surface expression of markers known to be upregulated on lung-resident CD4^+^ T cells, including CD69, CD49a, CD103, CXCR6, and CXCR3 (Fig. 3a). Among the total Tmem cell populations, all groups receiving BCG prime demonstrated an increase in CXCR6 as well as a moderate increase in CD69. As shown in Fig. 3b increased expression of surface markers CD69^+^, CD49a^+^, and CXCR3^+^ was observed only in lungs of mice boosted with KFE8-Ag85B-pulsed DCs compared with all groups. Expression of CD103 among the total Tmem populations did not vary with treatment group (Fig. 3b).

We utilized the approach illustrated in Fig. 2b, and used intravascular (i.v.) anti-CD45 staining to discriminate between vascular and parenchymal populations identified as CD45^+^ and CD45^−^ respectively^28^. A nanofiber dependent effect on Trm phenotype marker expression was more apparent among the parenchymal CD4^+^ Tmem cells (Fig. 3c), as compared to the total (Fig. 3b). Increased expression of CD69, CD103, and CXCR3 was observed in both the KFE8-Ag85B and the KFE8-Ag85B-pulsed DC groups compared with BCG vaccinated and unvaccinated controls (Fig. 3c), however, there was no statistical significance in the difference in percentage of CXCR3^+^ or CXCR6^+^ parenchymal Tmem cells with or without DCs (Fig. 3c); although when compared with BCG only controls, CXCR3^+^ expression was significantly increased in both PNF-boosted groups. Interestingly, within the parenchymal Tmem population, CD49a expression was greatest in the KFE8-Ag85B-boosted group compared with all other groups (Fig. 3c). These data indicate that DC-based delivery of KFE8-Ag85B nanofibers appear to increase the overall proportion of parenchymal cells and cells expressing CD69 and CD103 Trm phenotype markers or cells undergoing transient activation^11^.

### High dimensionality analysis reveals KFE8-dependent shift in Trm surface marker profile of Ag85B-specific CD4^+^ T cells

**Fig. 4:**
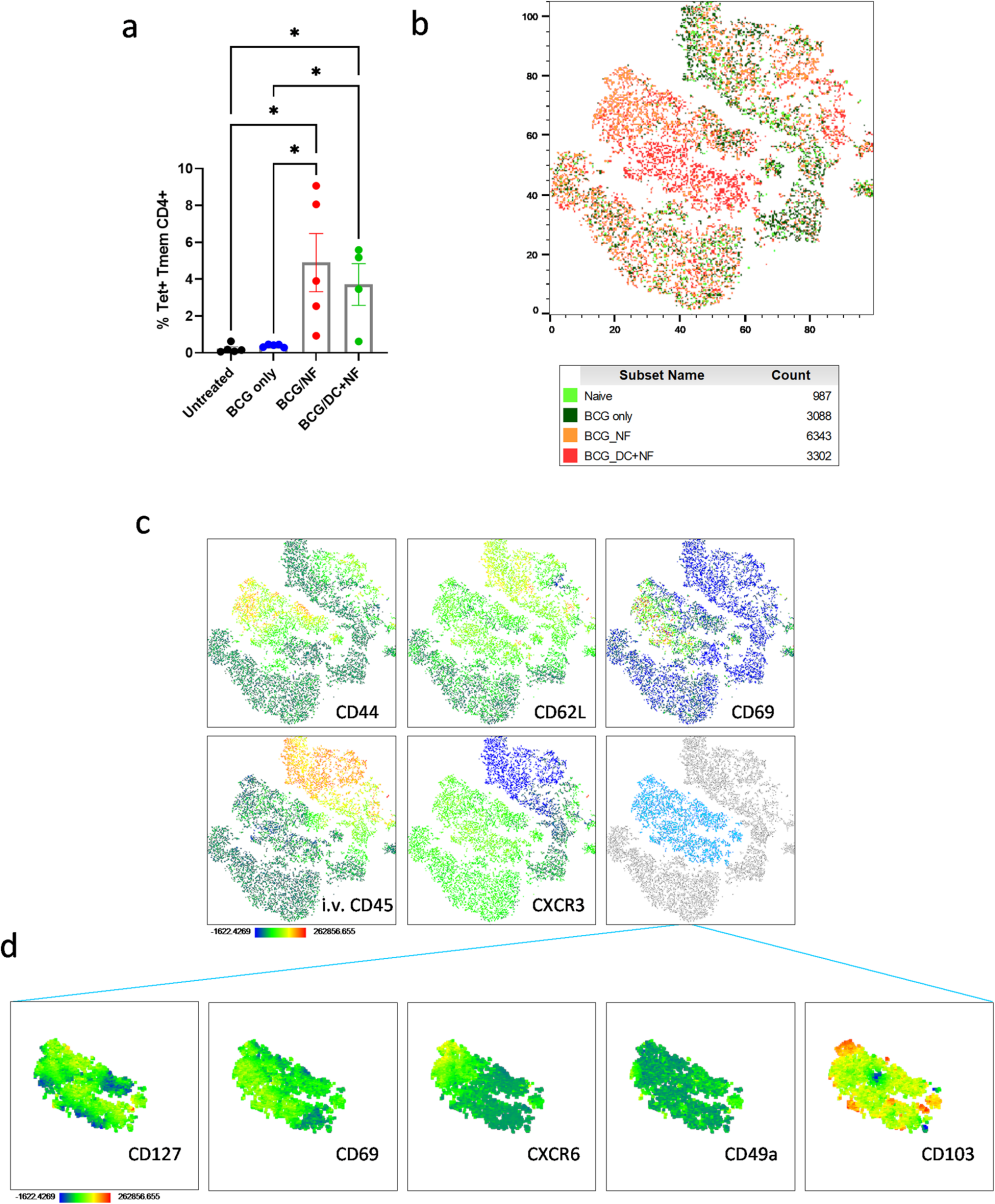
High dimensionality analysis reveals KFE8-Ag85B-dependent shift in Trm surface marker profile of Ag85B-specific CD4^+^ T cells in BCG-primed and boosted mice. **a** We gated for tetramer^+^ CD4^+^ T cells in each group. Data shown as mean ± SE. Significance was determined by one-way ANOVA followed by a Benjamini and Hochberg test. **b** The tetramer^+^ cells from each group were analyzed as a whole and visualized in a tSNE plot. The cells isolated from each treatment group are highlighted **c** and heatmaps were generated to describe intensity of specific markers CD44, CD62L, CD69, CD45, and CXCR3. Area highlighted in light blue represents the cell populations most augmented by KFE8-Ag85B and KFE8-Ag85B-pulsed DCs. **d** Heat maps are representative of light blue population and include markers CD127, CD69, CXCR6, CD49a, and CD103. Dark blue regions of heat maps correspond with lowest the lowest intensity of fluorescent antibody staining, whereas red corresponds with the highest intensity and ranges from −1622 up to 262856.

Intratracheal administration of KFE8-Ag85B and KFE8-Ag85B pulsed DCs consistently generated increased numbers of tetramer^+^ Tmem CD4^+^ T cells compared with untreated mice and those that only received BCG inoculation (Figs. 1f, 2c, 4a). Traditional flow cytometric analyses showed generation of parenchymal CD4^+^ T cells that recognize Ag85B and have increased expression of surface markers strongly associated with memory, tissue residency, and activation. To visualize the multidimensionality of the populations that make up the tetramer^+^ CD4^+^ T cells in the lung and identify shifts in population frequencies, tSNE (t-distributed stochastic neighbor embedding) plots were generated in FlowJo v10 using the default parameters and each experimental group was assigned a unique color (Fig. 4b). The tetramer^+^ CD4^+^ cells represent a diverse group of memory, effector, and recently activated T lymphocytes as shown in heat maps representing the intensity of surface markers CD44, CD62L, CD69, CD45, and CXCR3 (Fig. 4c). With these markers we identified a core collection of cells that exhibit a Trm-like phenotype (CD44^hi^ CD62L^lo^ CD69^+^ i.v.^-^ CXCR3^+^). We also observe a striking division between cells isolated from controls and the KFE8-Ag85B and KFE8-Ag85B-pulsed DC boosted groups highlighted in orange and red respectively (Fig. 4b). This central cluster of cells highlighted in light blue (Fig. 4c), is predominantly i.v.^-^ Tmem with a relatively high frequency of CD69^+^ cells and contained almost exclusively cells from mice boosted with KFE8-Ag85B or KFE8-Ag85B-pulsed DCs. When this group was further examined for additional markers of tissue-residency, we observed a heterogeneous population. Most cells represented in this grouping exhibit high intensity staining of CD127, as well as CD69. Less than half positively express CXCR6 and small populations of cells express CD49a or CD103 (Fig. 4d).

To determine if the addition of DCs specifically augmented the Trm phenotype of tetramer^+^ Tmem cells generated by KFE8-Ag85B boosting, we compared the expression of CD69 and i.v. staining between the two groups from two iterations of the study. We found that surface marker composition of tetramer^+^ cells was largely unchanged between the two groups (Supplemental Fig. 3a, b). To assess phenotypic changes unique to groups boosted with KFE8-Ag85B, tetramer^+^ Tmem cells from the KFE8-Ag85B boosted and KFE8-Ag85B pulsed DCs boosted groups were combined and compared to the total Tmem population from both groups. There were no differences between tetramer^+^ Tmem and total Tmem populations, with the exception of enhanced CD49a expression in the tetramer^+^ cells (Supplemental Fig. 3c).

Taken together, these data suggest that pulmonary boost with KFE8-Ag85B or KFE8-Ag85B-pulsed DCs generated a heterogeneous population of Ag85B-specific Trms, which primarily reside in the parenchyma, express important adhesion molecules, and tissue homing markers. Importantly, our results demonstrate that use of KFE8 bearing an epitope of choice generates Trm in the lung in the setting of BCG primed immunity. The adaptability of KFE8 for multivalent presentation of additional epitopes along with optimized adjuvant design could subsequently be exploited to generate and promote tissue resident status of additionally protective T cell populations.

### KFE8-Ag85B boost induces antigen-specific memory cytokine responses in splenic T cells

**Fig. 5:**
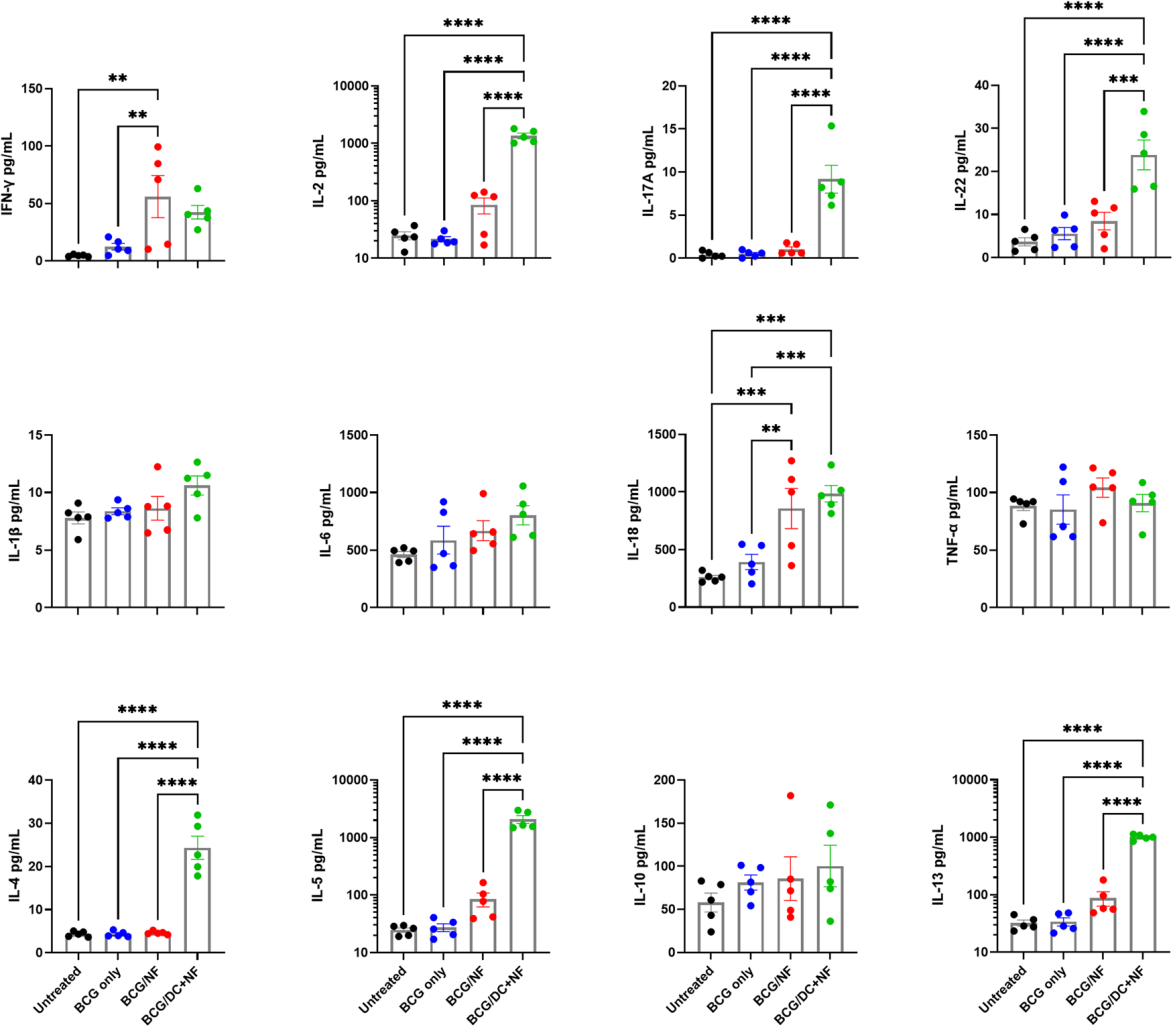
KFE8-Ag85B boost in BCG-primed mice induces antigen-specific memory cytokine responses in splenic T cells. Four weeks post boost, splenocytes from animals in each treatment group (*n* = 5) were co-cultured in a 10:1 ratio with BMDCs pulsed overnight with cognate antigen (Ag85B_240–254_). Cytokine production by splenocytes after 72 h of co-culture was analyzed by multiplex ELISA. Data shown represent Th1 (IFN-γ, IL-2), Th17 (IL-17A, IL-22), Th2 (IL-4, IL-5, IL-13), pro-inflammatory (IL-1β, IL-6, IL-18, TNF-α), and regulatory (IL-10) cytokines. Data shown as mean ± SE. Significance was determined via one-way ANOVA followed by a Benjamini Krieger Yekutieli post hoc test for multiple comparisons to determine differences due to treatment and among treatment groups. *p < 0.05, **p < 0.01, ***p < 0.001, ****p < 0.0001.

To determine if circulating memory CD4^+^ T cells responsive to cognate antigen were generated with immunizations, we measured cytokine release with an ex vivo recall assay using splenic T cells from vaccinated mice. Briefly, DCs were pulsed overnight with antigen (Ag85B peptide) or PBS, overlaid with splenic T cells in a 10:1 ratio, and co-cultured for 72 h. Supernatants were collected and analyzed for cytokine production using a multi-plex ELISA. Key cytokines (Th1,Th17, Th2, pro-inflammatory) produced in response to cognate antigen are shown in Fig. 5. Splenocytes from the group boosted with KFE8-Ag85B pulsed DCs produced the highest amount of several cytokines (e.g., IL-2, IL-17A, IL-22, IL-4, IL-5, and IL-13) in response to Ag85B; whereas, the splenocytes from the KFE8-Ag85B boosted group produced the most IFN-γ and similar amounts of IL-18. Production of IL-1β, IL-6, IL-10, and TNF-α did not significantly change between treatment groups (Fig. 5), although a nonsignificant increase in IL-10 was observed in the group boosted with KFE8-Ag85B pulsed DCs. Cytokines and chemokines with results below baseline or lacking statistical significance are not shown.

Splenocytes from the same samples were collected after antigen recall and processed for intracellular staining to characterize groups of cytokine-producing T cells^29^, including polyfunctional CD4^+^ T cells using combinatorial Boolean analysis. The number of cells in lung tissue were too few for flow cytometric analysis of both memory subpopulations and intracellular cytokine recall; however, spleen provided sufficient cells numbers (Supplemental Figs. 4, 5). There were no significant differences in cells singly producing IFN-γ, IL-2 or TNF-α between treatment groups, although moderate increases in IL-2 and IFN-γ were observed in the mice boosted with KFE8-Ag85B or KFE8-Ag85B pulsed DCs, respectively. However, splenocytes from mice boosted with KFE8-Ag85B pulsed DCs demonstrated a significant increase in IL-17A compared with all other groups, and mice boosted with KFE8-Ag85B exhibited a significant reduction in IL-17A compared with controls (Supplemental Fig. 4a). When all cytokine-producing CD4^+^ T cells were visualized as a whole, shifts in IL-2^+^ T cells due to KFE8-Ag85B, and shifts in IL-17A^+^ and polyfunctional T cells due to KFE8-Ag85B pulsed DCs, become more pronounced (Supplemental Fig. 4b).

Mice boosted with KFE8-Ag85B pulsed DCs demonstrated a significant increase in overall polyfunctionality, an effect that reached significance compared with BCG alone (Supplemental Fig. 5a). These results are also presented as stacked columns to quantify populations of CD4^+^ T cells that express two, three, or four cytokines (Supplemental Fig. 5b). CD4^+^ T cells from mice vaccinated with KFE8-Ag85B pulsed DCs exhibited an increased frequency of triple cytokine expression compared with all other groups. Interestingly, animals that received a BCG prime only or a KFE8-Ag85B boost demonstrated a reduction in CD4^+^ T cells expressing three cytokines compared with untreated controls or vaccinees receiving a KFE8-Ag85B pulsed DC boost (Supplemental Fig. 5c). T cells were further characterized for polyfunctional cytokine signatures, as visualized in Supplemental Fig. 5c. In all groups, regardless of treatment, IL-2^+^ IFN-γ^+^ IL-17A^+^ T cells are a dominant polyfunctional profile, followed by IL-2^+^ TNF-α^+^ and IL-2^+^ IL-17A^+^ T cells. An increased number of IL-2^+^ IFN-γ^+^ T cells were also observed in groups primed with BCG and treated with either mock or KFE8-Ag85B boost (Supplemental Fig. 5d).

**Fig. 6:**
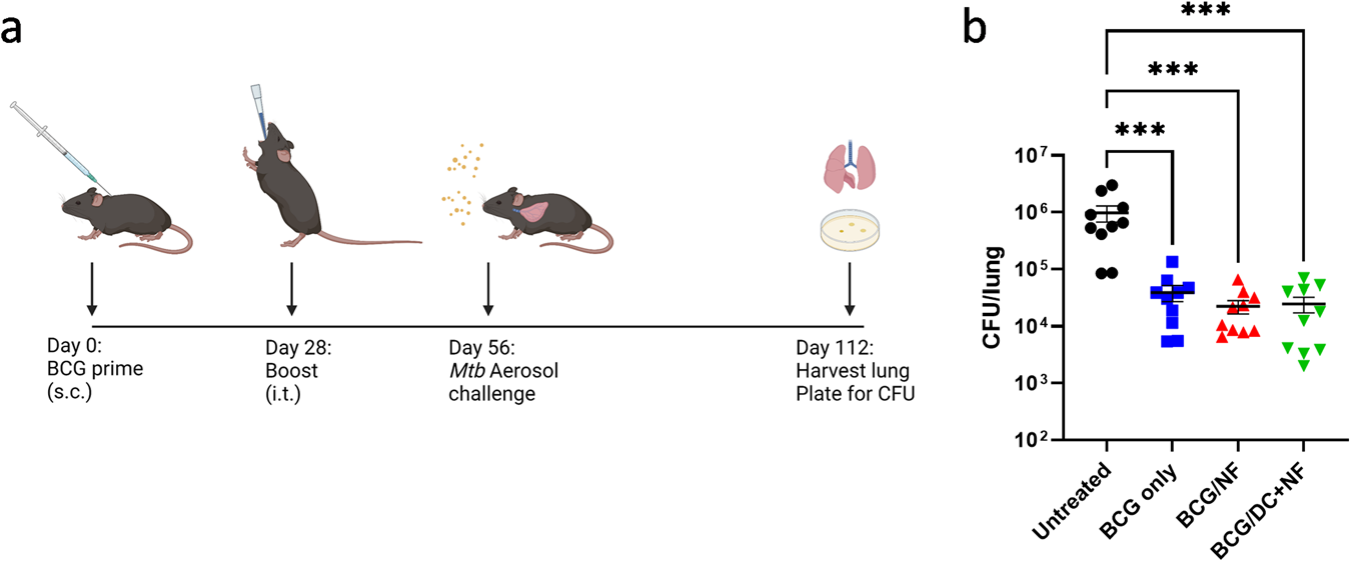
Pulmonary administration of KFE8-Ag85B or KFE8-Ag85B pulsed BMDCs does not significantly improve BCG-induced protection. **a** BCG-primed mice were given i.t. boosts four weeks later with either KFE8-Ag85B or KFE8-Ag85B pulsed DCs. An additional four weeks after boost, mice were challenged via an aerosol route with 100 CFU of *Mtb* H37Rv. **b** Eight weeks p.i. lungs were harvested, homogenized, and plated for CFU enumeration. Data shown as mean ± SE. Significance was determined by one-way ANOVA followed by a Benjamini Krieger Yekutieli post hoc test for multiple comparisons to determine differences due to treatment and among treatment groups. *p < 0.05, **p < 0.01, ***p < 0.001, ****p < 0.0001.

To determine if boosting BCG-primed mice with KFE8-Ag85B or KFE8-Ag85B pulsed DCs provides additional protection compared to BCG, mice were challenged with virulent *Mtb* via the aerosol route at 4 weeks post-boost (Fig. 6a). At 8 weeks post aerosol infection, a significant decrease in lung mycobacterial load of greater than 1 log was observed in mice that received a prime dose of BCG. Mice boosted with KFE8-Ag85B or KFE8–85B pulsed DCs, however, did not display additional reduction in lung bacterial burden compared with BCG prime alone (Fig. 6b).

Overall, these findings indicate that KFE8-Ag85B-pulsed DCs drive Th1, Th17, and Th2 recall responses to Ag85B in BCG-primed mice. Further investigation to determine early responses in lung tissue should be pursued. Boosted mice did not exhibit enhanced protection following challenge. These results are consistent with previous demonstrations that monovalent Ag85B-based subunit vaccines do not enhance BCG-generated protective immunity^30,31^.

### Transcriptional profiling reveals a KFE8-dependent immune activation signature

**Fig. 7:**
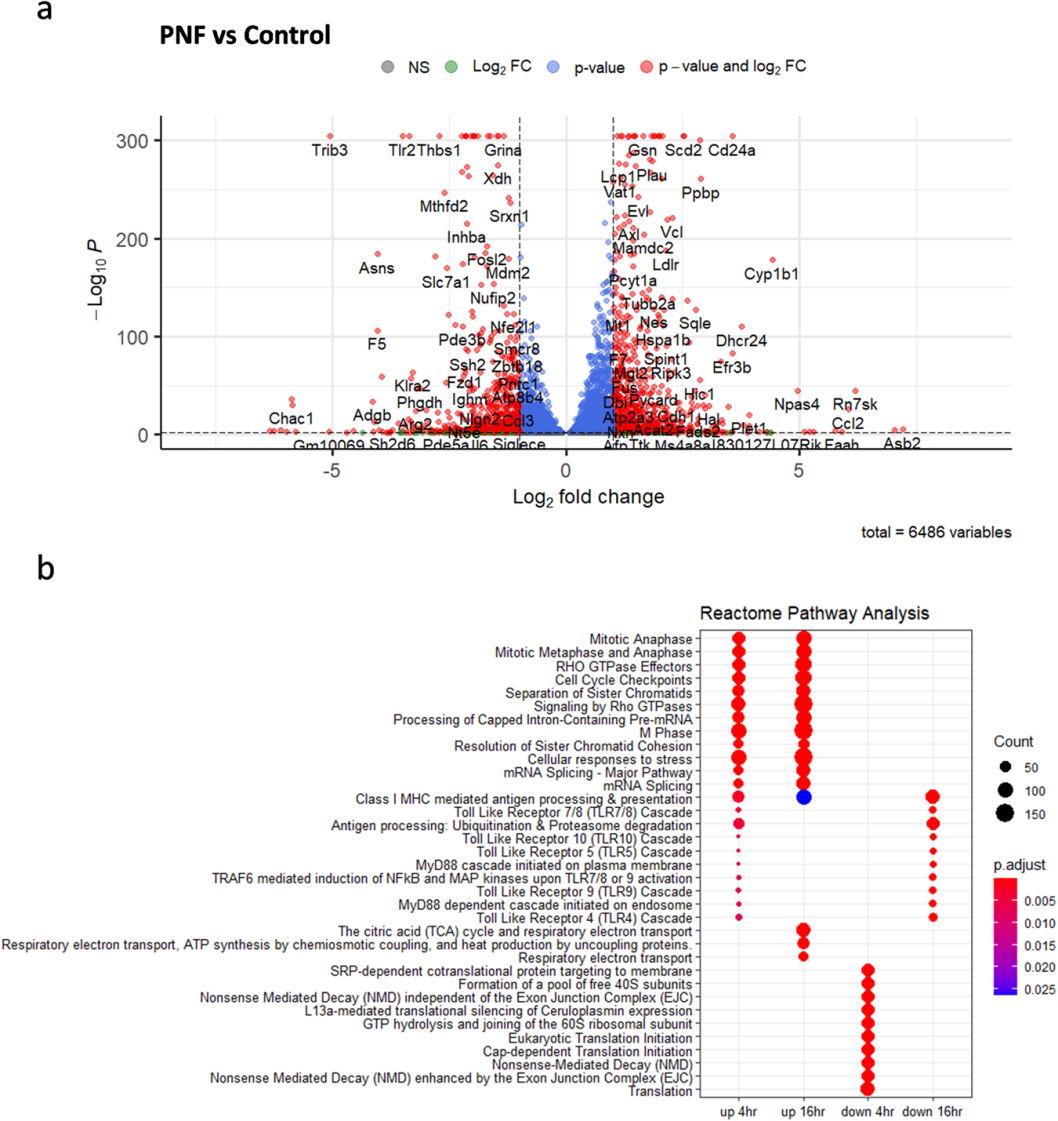
Transcriptional profiling of DCs in vitro reveals a novel immune activation signature of KFE8. RNA was extracted from 1 × 10^6^ DCs per sample 4 and 16 h after treatment with 10 µM KFE8 or with PBS (*n* = 2). **a** Transcriptomic changes between KFE8-treated cells and controls after 16 h were revealed through differential expression analysis and visualized in a volcano plot with cut-offs at log2 fold change of 1 or −1 and *p*-value of < 0.01. **b** Pathway enrichment analysis of the differentially expressed genes was performed using the ReactomePA package and clusterProfiler. The plot represents the top up- and down-regulated pathways and biological processes in both time points.

In order to identify potential pathways through which KFE8 may act to promote antigen presentation, activation and differentiation of T cells, we performed RNA sequencing of DCs treated with KFE8 or mock after 4 h and 16 h of exposure. Differential gene expression analysis revealed a total of 6,486 genes differentially upregulated or downregulated by KFE8 compared with control (Fig. 7a). The most highly upregulated pathways, as identified through pathway analysis using the Reactome PA package, include *Mitotic Metaphase and Anaphase*, *Cell Cycle Checkpoints*, *Separation of Sister Chromatids*, and *Resolution of Sister Chromatid Cohesion*. Several of the genes in these pathways were involved in regulation of the cell cycle and mechanisms of DNA repair. Additionally, *Cellular responses to stress*, *Class I MHC mediated antigen processing & presentation* were upregulated, further supporting our previous findings that KFE8 promotes antigen presentation^25^. Interestingly, a small set of genes involved in TLR signaling (e.g., *Toll like Receptor 10 Cascade, Toll Like Receptor 5 Cascade, TRAF6 mediated induction of NFκB and MAP kinases upon TLR7/8 or 9 Activation*) were upregulated after 4 h of KFE8 exposure and were significantly downregulated at 16 h of exposure. Pathways involved in translation (e.g., *SRP-dependent cotranslational protein targeting to membrane*, *Formation of a pool of free 40* S subunits, *Eukaryotic Translation Initiation*) were downregulated in the DCs exposed to KFE8 for 4 h; however, these levels return to baseline expression in later time points. After 16 h of KFE8 treatment, pathways involved in metabolic respiration, including *The tricarboxylic acid (TCA) cycle* and *Respiratory electron transport*, as well as *ATP synthesis* were upregulated, indicating a possible metabolic shift in the DCs (Fig. 7b). Together, these data suggest that KFE8 induces acute stresses on DCs resulting in halting of translation, triggering degradation and processing of antigen, and changes to mitochondrial metabolism. However, further exploration and validation of these results at the protein and cellular level will be required to accurately pinpoint the mechanisms through which KFE8 elicits its self-adjuvanting effects.

These data notably lack the strong inflammatory signals as would be expected in response to commonly employed adjuvants^32,33^. Overall, the results identify novel pathways activated by KFE8 that promote generation of memory CD4^+^ T cells that recognize cognate antigen and have a lung tissue-resident phenotype. The lack of activation of pathways elicited by traditional adjuvants indicates potential opportunities for greater expansion and/or increased functionality through combination constructs containing both KFE8 and adjuvants.

## Discussion

Successful development of effective booster vaccines for TB has long been hampered by challenges to generating cell-mediated immune memory that will protect the lung mucosa. Growing evidence in both murine and NHP models suggest that *Mtb*-specific CD4^+^ T cells with a lung-resident phenotype play an important role in protective immunity. Exploiting this knowledge for vaccine development, however, requires approaches that will safely complement or amplify lung resident memory T cells specific to *Mtb*. Approaches that can generate new, or boost pre-existing, Trm in persons with previous BCG vaccination history would have the most application. In recent years, natural and synthetic nanomaterials have been explored for the development of vaccines aimed at generating or boosting pulmonary Trm cellular immunity^34,35^. Here we demonstrate a self-adjuvanting PNF-based vaccine platform that effectively targets the lung mucosal immune response of BCG primed mice and amplifies a heterogenous population of CD4^+^ Trm specific to a targeted *Mtb* epitope.

Nanoparticles have been shown as effective for boosting memory at mucosal surfaces^18,19,23,36,37^ including immune responses to *Mtb* antigens^38^. Pulmonary delivery to the lung mucosal immune systems is expected to be an important immunization strategy to boost immunity in those with previous BCG intradermal vaccination^39–41^. The utility of PNFs as subunit vaccines has been previously demonstrated, including our previous report showing that nanofibers composed of the self-assembling peptide KFE8 used here and the TB10.4 epitope of *Mtb* induced generation of CD8^+^ T cell memory and activation of protective cell-mediated immunity^26^. In those experiments, we further observed that pulmonary delivery of co-assembled nanofibers with both TB10.4 and Ag85B also led to generation of CD4^+^ T cells with antigen-specific cytokine function, but did not improve protection against *Mtb* challenge in mice lacking a BCG prime^26^. In support of these previous observations, pulmonary KFE8-Ag85B immunization in the current study generated robust populations of effector and memory CD4^+^ helper T cells in the lung, including those with binding specificity to an Ag85B-bearing tetramer. Comparative analysis of the activated and memory CD4^+^ T cell populations in the lung and spleen confirmed that pulmonary delivery of KFE8-Ag85B effectively targets the lung mucosal immune system and enhances the immune response in lungs of mice previously immunized with BCG.

Our comprehensive analysis of cellular phenotype demonstrates that KFE8-Ag85B boost generated robust populations of CD4^+^ Trm in the lung parenchyma. These results included the expected changes to surface markers CD44, CD62L, and CD69, which are frequently used to identify CD4^+^ and CD8^+^ Trms in both mice and humans^42,43^. However, CD69 is also a marker of early activation and is not always sufficient for the formation of CD4^+^ Trms^44–46^. Inclusion of an extended array of surface markers associated with tissue-residency, including CD103, CD49a, CXCR3, CXCR6, and CD45 for i.v. staining^28,47,48^ conclusively identified Trm populations generated by the KFE8-Ag85B boost. The parenchymal populations were almost exclusively CXCR3^+^ as compared to the vascular compartment where terminally differentiated KLRG1^+^ CD4^+^ T cells have been shown to reside^40,49^. These results are consistent with other observations^13,28^ and provide additional validation for tissue residency. Importantly, a high proportion of the antigen-specific (tetramer^+^) CD4^+^ T cells in the parenchymal compartment also displayed an increased frequency of tissue residency markers compared with the vascular compartment. In short, boosting with KFE8-Ag85B promoted an increase in *Mtb*-specific CD4^+^ T cell populations that are associated with rapid response function due to their localization in the lung parenchyma.

The Ag-specific Trm populations in the lung observed following KFE8-Ag85B boost displayed a surprising degree of heterogeneity, especially with regards to differential expression of CXCR6, CD49a, and CD103. The immunological significance of Trm heterogeneity is incompletely understood to date and likely reflects different functional attributes. CXCR6 is expressed by activated T lymphocytes, directs cells into sites of inflammation, and is thought to play a role in maintaining tissue resident populations^50–52^. Expression of CD49a or CD103 has been shown to be less uniform among the CD4^+^, compared to the CD8^+^, Trm populations^41,42^. Functional roles for these two markers have been described for CD8^+^ Trms. CD49a binds to collagen IV, which in the setting of influenza infection has been shown to mediate migration of CD8^+^ Trms. In the same infection model, inhibiting the interaction of CD103 with its ligand E-cadherin increased CD8^+^ T cell motility, suggesting a role in cell retention^53^. We observed that CD4^+^ parenchymal Tmem populations in lungs of mice boosted with KFE8-Ag85B exhibited greater expression of CD49a, and those boosted with KFE8-Ag85B-pulsed DCs expressed significantly more CD103. These findings demonstrate that pulmonary boost with a PNF vaccine displaying an *Mtb* epitope of choice can activate a pool of Trm with potentially diverse localization, trafficking, and immune function.

Peripheral cell-mediated immunity was also boosted in response to pulmonary boost with KFE8-Ag85B. Cytokine recall of splenocytes indicated the presence of memory cells with Th1 (e.g., IFN-γ, IL-2) and Th2 (e.g., IL-5, IL-13) differentiation bias. This outcome was especially prominent in response to pulsed DC immunization, which also activated Th17 family cytokine (e.g., IL-17 and IL-22) recall. An unexpected outcome was the antigen-specific increase in IL-18 in vaccinated animals. IL-18 is not a typical T cell cytokine, but is produced by a variety of other cells including antigen-presenting cells, and functions, in part, to induce IFN-γ production in T cells^54,55^. Lack of evidence for significant pro-inflammatory cytokine (e.g., IL-1β, IL-6, TNF-α) activation reduces the probability that cytokine responses to antigen were driven by bystander effects. Similarly, the modest pro-inflammatory cytokine responses observed due to PNF in our studies support the lack of lung damage reported in previous studies utilizing pulmonary delivery of PNFs^37^. These findings demonstrate the development of moderate cell-mediated immunity in the peripheral immune system following KFE8-Ag85B vaccination. Delivery of KFE8-Ag85B-pulsed DCs further activated peripheral memory cells, including an enhanced cytokine production and an expanded effector cytokine profile in response to the targeted vaccine epitope.

Previous investigations by our group demonstrated that KFE8 nanofibers are self-adjuvanting and enhance antigen presentation through proteasome- and autophagy-dependent mechanisms^25^. The DC-dependent effects observed in our current study suggest an advantage of immune stimulation beyond the self-adjuvant capacity. Consistent with our findings, adoptive transfer of adjuvanted DCs was previously shown to augment BCG-induced immunity against *Mtb* by overcoming the antigen presentation bottleneck^56,57^. Many vaccination strategies targeting T cell immunity have utilized techniques to improve DC function and antigen presentation through PRR stimulation, reinfusion of antigen-loaded ex vivo, or in vivo targeting of DCs. Anti-cancer therapeutics that employ DC targeting methods generate cancer immunity by inducing tumor-specific Th1 and cytotoxic T cells^58^. DC-based approaches are less widely studied for preventing infection due to limitations for clinical translation in resource-limited settings. The significance of our findings is thus an important proof-of-concept that the immunogenicity of Ag85B PNFs can be markedly enhanced in the setting of optimized antigen presentation.

The expanded pools of *Mtb*-specific Trm cells within the lung parenchyma of KFE8-Ag85B-boosted and KFE8-Ag85B-pulsed DC-boosted groups did not translate to improved protection against a robust aerosol challenge with *Mtb*, compared with BCG alone. This was not an unexpected result as our vaccine design contained only one *Mtb* antigen and was designed to show a proof-of-concept that KFE8 could augment CD4^+^ Trm populations in a tractable system. Ag85B has well-described immunogenicity^59,60^ and has been integrated into several vaccine strategies, several of which are in clinical trials^61^. Despite being an important immunogen, Ag85B-specific T cells are markedly contracted 4 weeks after infection^62^ due to waning antigen availability and is thus often included among an expanded repertoire of antigens. While Trm cells are often thought to concentrate at the site of prior vaccination or infection, emerging evidence suggests that they can disseminate throughout the body^63^. Studies examining the relative effectiveness of disseminated versus localized Trm populations suggest that the site of T cell priming is not crucial, but local inflammation and repeat antigen exposures enhance Trm accumulation and retention, thereby providing optimal protection^64^. Our studies utilized a single Ag85B nanofiber boost and highlight the limitations of Trm generation and accumulation. Thus, multiple and multi-epitope boosters may provide potent pathogen control.

Transcriptome analysis of differential gene expression in the current study showed that KFE8 exposure in APCs drives marked upregulation of many genes involved in cellular responses to stress, mitochondrial respiration, DC migration and maturation, and detoxification or antioxidant functions. Additionally, evidence of upregulated antigen processing in KFE8-treated cells supports our previous findings that KFE8 enhanced antigen presentation through mechanisms of autophagy^25^. These findings suggest that the self-adjuvanting effects of KFE8 are, in part, driven by exposure to DAMPs that result from contact with KFE8 nanofibers after a pulmonary delivery. Activation of immune responses by DAMPs have been shown to improve cell-mediated immune responses and increase Trm functionality following mucosal vaccination with recombinant adenoviral vectors containing influenza antigens and vector-encoded IL-1β^65^. Similarly, utilization of HMGB1 as an adjuvant in an influenza vaccine demonstrated improved T cell responses in the lung^66^, and serve as endogenous TLR activators^67^. Interestingly, genes that regulate common components of several TLR pathways were upregulated at 4 h, including mitogen-activated protein kinases (*Mapkapk2*, *Map2k3*, *Map2k4*, and *Map3k7*), activator protein I (*AP-1*) family members (*Jun* and *Fos*), NFkB components (*Nfkb1* and *Nfkbia*), *Atf1*, and *Ikbkg* among others. Several of these TLR activation-related genes were subsequently downregulated after 16 h, as were Traf6 and IL-1 receptor kinases (Irak1 and Ikrak2). These results indicate that KFE8 may modulate TLR signaling through DAMPs and support our previous observations that KFE8 moieties activate antigen processing and presentation through proteasomal processes^25^. Consistent with our in vitro observations (Fig. 5), we did not observe significant changes in pro-inflammatory cytokine mRNA transcripts. It is important to note that the overall profile of genes activated by KFE8 markedly differed from that previously observed with BCG^68^ or common adjuvants in APCs, which often include strong pro-inflammatory (e.g., NFκB) and other PRR pathways (e.g., TLR) or IFN signaling predominates^33^, which were not observed in abundance in our transcriptomic study of KFE8-treated DCs. Further assessment and validation of the APC response to KFE8 are critical for efforts to fully optimize immune responses to PNF vaccines and similar constructs at the time of vaccination.

In conclusion, our work demonstrates the utility of the KFE8 nanofiber construct as a subunit vaccine platform in generating a robust expansion of CD4^+^ Trm populations in the lungs of BCG-primed mice. Current paradigms in the field support a need for multivalent TB vaccines that generate immune protection from both CD4^+^ and CD8^+^ T cells^69,70^, as well as from functional B cells and other immune cell populations^71^. Future iterations would build on our current findings to include other immunogenic *Mtb* antigens and adjuvants. Studies from our lab and others have demonstrated that whole proteins can be conjugated or admixed with PNFs using orthogonal chemistries or biotic expression^72–74^. Due to the modular nature of self-assembly, stoichiometric ratios of epitopes can be controlled to elicit optimal immunity. Given the richness of chemical functionality available in peptides, orthogonal chemistries can be developed for conjugating immunopotentiators (e.g., TLR agonists) for improving memory responses^26^. Importantly, the TLR agonist concentration can be titrated to ensure maximal immunogenicity without overt inflammation^75^. The synthetic nature of PNFs allows for high purity, minimal contamination, and mass-spectrometry based validation of the final product—a significant advantage over current emulsion/extract adjuvants for their regulatory evaluation. In sum, PNFs are an attractive platform for designing subunit BCG-booster vaccines with optimized antigen and adjuvant compositions to augment lung Trms.

## Methods

### NF synthesis and preparation

Peptide synthesis was performed on a CSBio 336 automated synthesizer using standard Fmoc chemistry and crude peptide (FKFEFKFE-GGAAY-FQDAYNAAGGHNAVF) was purified using a water/acetonitrile gradient on a Varian HPLC system^26^. Purity of peptide was assessed by HPLC and identity was confirmed using MALDI-TOF spectroscopy. KFE8-Ag85B stocks were prepared by resuspending lyophilized KFE8-Ag85B in sterile, endo-toxin free water in a 1 mM solution, and working stocks were made by diluting the stock in sterile PBS to bring the solution to 0.1 mM.

### Bacteria and growth conditions

BCG Pasteur (ATCC 35734) was propagated using Middlebrook 7H9 media supplemented with 10% v/v OADC (oleic acid, albumin, dextrose, catalase), 0.05% Tween80 v/v, and 0.5% v/v glycerol. After thaw, BCG was propagated in 5 mL of culture media in a 37 °C incubator. Cultures were grown to an optical density (OD_600_) of 0.4–0.6 prior to use, after which the cultures were pelleted and washed two times in PBS for use in vaccinations. Pellets were resuspended in sterile PBS at a final density of 5.5 x 10^6^ colony-forming units per 1 mL (CFU/mL). The bacterial suspensions were plated on petri plates containing Middlebrook 7H10 supplemented with 10% OADC v/v and 0.5% v/v glycerol for CFU enumeration in 10-fold serial dilutions. All experiments with BCG were conducted under BSL2 or ABSL2 conditions with appropriate safety equipment and protocols in place. *Mycobacterium tuberculosis* H37Rv (ATCC 25618) was similarly propagated. All studies with *Mtb* were conducted in Biosafety level 3 (BSL3) or animal BSL3 (ABSL3) facilities following established guidelines and safety protocols as approved by the UTMB Environmental Health and Safety Division of Biosafety.

### Immunization and challenge

All animals in this study were female C57BL/6 mice supplied by Jackson Labs at approximately 6–8 weeks old and housed in either ABSL2 or ABSL3 facilities. All experiments employing use of mice were approved by the University of Texas Medical Branch Institutional Animal Care and Use Committee. Mice were given ad libitum access to food and water throughout the duration of all studies. Mice were first given subcutaneous (s.c.) vaccinations of 5.5 × 10^5^ CFU BCG in 100 µL of sterile PBS. 4 weeks post prime, mice were given an intratracheal (i.t.) boost under 1–3% isoflurane anesthesia with 100 µL of sterile PBS containing either 100 µM PNF, 5 × 10^5^ BMDCs pulsed with 100 µL of 100 µM PNF, or PBS (*n* = 4–5/group or *n* = 10/group). BMDCs were prepared from bone marrow of age-matched female C57BL/6 mice. Femur and tibia bones were flushed and subsequently cultured at a concentration of 1 × 10^6^/mL in RPMI-1640 containing 10% FBS, and penicillin-streptomycin. The culture was supplemented with 10 ng/mL of mouse recombinant GM-CSF (STEMCELL, 78017.1) and 10 ng/mL of recombinant mouse IL-4 (STEMCELL, 78047.1). Additional media containing rGM-CSF and rIL-4 was added after 3 days and 6 days of culture. Floating cells were harvested after 7 days of culture and plated at 1 × 10^6^ cells in each well of a 24 well plate. Prior to boosting, BMDCs were pulsed overnight with 100 µM of PNF per mouse. After an additional 4 weeks post boost, mice in some studies were challenged with *Mtb* via an aerosol route using the Biaera Technologies computer-controlled aerosol regulation system with the assistance of the UTMB Aerobiology Service Core in ABSL3 facilities. *Mtb* suspensions were prepared from logarithmic growth phase cultures in 10 mL of Middlebrook 7H9 media + OADC + Tween80 containing 0.1% anti-foam v/v (Sigma, St. Louis, MO)^26^ and used to deliver a calculated dose of 100 CFU per mouse.

### CFU enumeration

BCG cultures used for immunizations were enumerated by plating 10-fold serial dilutions in sterile PBS on 7H10 media supplemented with OADC followed by 3 weeks of incubation at 37 °C^26^. Following aseptic removal, lung tissue was collected for CFU enumeration after *Mtb* challenge. Tissue was homogenized in 1 mL of sterile PBS, serially diluted in 10-fold increments in sterile PBS and plated on 7H10 media supplemented with OADC for 3 weeks at 37 °C prior to enumeration of bacterial colonies.

### Tissue processing and flow cytometry

In the experiments described in Fig. 1, lungs were perfused with sterile PBS after euthanasia by slowly pushing approximately 10 mL of sterile PBS through the right ventricle of the heart. Lung tissue was minced and incubated in 3 mL of serum-free media containing 0.5 mg/mL DNase I (Roche, 10104159001) and 1 mg/mL collagenase type IV (Worthington, LS004188) in a 37 °C humidified incubator for 45 min. In studies performed in Figs. 2–5, mice were given tail-vein injections of 1.5 µg of BV711 anti-CD45 (BD Biosciences, 563709) antibody three minutes prior to euthanasia to differentiate between parenchymal (CD45−) and vascular (CD45+) lymphocytes^28^. Then lungs were removed, minced, and rinsed several times before enzymatic digestion. The minced and digested tissue was then poured over and pressed through a 70 µm cell strainer on a 50 mL conical tube containing 10 mL of complete cell culture media (RPMI + 10% FBS + 1x Pen/Strep). Cells were pelleted at 300 × *g* for 5 min, then resuspended in 1 mL of RBC lysis buffer (Sigma, R7757–100ML) for 1 min before stabilization with sterile PBS and centrifugation. The lung cells were resuspended in complete RPMI and centrifuged at 60 × *g* for 1 min. The supernatant containing leukocytes was carefully transferred to a new 50 mL conical. This was repeated two additional times. Splenocytes were isolated by passing the organ through a 70 µm cell strainer into a 50 mL conical containing complete RPMI. Cells were isolated, resuspended in complete RPMI, and counted following RBC lysis and washing.

The lung cell suspensions and 1 × 10^6^ splenocytes for each mouse were pelleted at 300 × *g* for 5 min and resuspended in 1 mL of PBS containing 0.1% v/v Fixable Viability Dye eFluor 506 (eBioscience, 65–0866–14). Cells were incubated at 4 °C for 15 min, washed in PBS, and incubated with Fc block (BD Bioscience, 553142) in cell staining buffer (1% BSA Fraction V w/v, 0.1% NaN_3_ w/v in calcium- and magnesium-free PBS) for 5 min. To identify Ag85B-specific T cells and characterize cellular phenotype, cells were stained for 1 h at 4 °C with an MHC-II Ag85B tetramer obtained through the NIH Tetramer Core Facility (I-A(b) FQDAYNAAGGHNAVF), followed by an additional hour of staining at 4 °C with anti-CD3 (BUV395, BD Biosciences, 563565, 1 µL per test), anti-CD4 (BUV496, BD Biosciences, 612952, 1 µL per test), anti-CCR7 (PE-Dazzle 594, Biolegend, 120122, 2.5 µL per test), anti-CD44 (PE-Vio770, Miltenyi Biotec, 130–102–377, 2.5 µL per test), anti-CD62L (Brilliant Violet 786, BD Biosciences, 564109, 1 µL per test), anti-CD69 (APC, Biolegend, 104514, 2.5 µL per test), anti-CD49a (VioBright FITC, Miltenyi Biotec, 130–107–592, 2.5 µL per test), anti-CD103 (Brilliant Violet 605, BD Biosciences, 748257, 1.25 µL per test), anti-CD127 (Brilliant Violet 650, BioLegend, 135043, 1.25 µL per test), anti-CXCR3 (PerCP-Cy5.5, BioLegend, 126514, 0.625 µL per test), and anti-CXCR6 (PE, BioLegend, 151104, 1.25 µL per test) antibodies. Lung samples contained on average 5 × 10^6^ cells per mouse. Cells were washed in cell staining buffer and fixed in 2% ultra-pure formaldehyde (Polysciences, 18814–10) prior to acquisition.

To identify and characterize polyfunctional CD4^+^ T cells, splenocytes were treated with GolgiStop (BD Biosciences, 554724) 5 h prior to staining. Supernatants were transferred to separate tubes and used as described above for soluble cytokine analysis, and cells were collected and washed in sterile PBS. Following viability and surface staining, splenocytes were permeabilized and fixed in BD Cytofix/Cytoperm (BD Biosciences, 554722) as per manufacturer’s instructions. Cells were then washed with BD Perm/Wash Buffer (BD Biosciences, 554723). The antibody cocktail was prepared in BD Perm/Wash Buffer, including IFN-γ (Brilliant Violet 605, Biolegend, 505840, 0.625 µL per test), TNF-α (APC, Biolgened, 506108, 0.625 µL per test), IL-2 (Brilliant Violet 711, Biolegend, 503837, 1.25 µL per test), and IL-17A (PE, Biolegend, 506904, 0.625 µL per test) and cells were stained for 1 h at 4 °C. Splenocytes ranged from 1 × 10^6^ cells to 2 × 10^6^ cells per sample. Splenocytes were washed in BD Perm/Wash Buffer for a final time and resuspended in 2% ultra-pure formaldehyde prior to acquisition.

Acquisition of samples was performed using BD LSRII Fortessa flow cytometer at the UTMB Flow Cytometry and Cell Sorting Facility. The compensation matrix was developed using UltraComp ebeads (Invitrogen, 01–2222–42) and calculated using the BD FACSDiva software. The analysis of cell phenotypes was performed using FlowJo v10 software. To generate tSNE plots, tetramer^+^ CD4^+^ CD3^+^ cells from each treatment group including controls were concatenated into 4 individual.fcs files, then concatenated once more into a single.fcs file, with each assigned a parameter to define the treatment groups. tSNE plots were generated using CD44, CD62L, CD45, CD127, CD69, CD103, CD49a, CXCR3, and CXCR6 as parameters and default settings in FlowJo v10. The Boolean analysis function in FlowJo was used to characterize and quantify populations of polyfunctional CD4^+^ T cells^29^.

### Antigen recall

Eight days prior to necropsy, bone marrow was collected from age-matched C57BL/6 mice and treated with mouse rGM-CSF and rIL-4. The day prior to necropsy, BMDCs were harvested and plated at a density of 1 × 10^6^ per well in a 24 well plate with 10 µM cognate antigen (Ag85B_240–254_) or with vehicle control (PBS) in complete RPMI. Fresh splenocytes from vaccinated animals were overlaid on antigen-pulsed BMDCs in a 10:1 ratio. The number of T cells obtained from disrupted lung tissue at 4 weeks post-boost was insufficient in number to utilize for antigen recall, therefore, splenocytes were used to assess the memory recall due to vaccination. After 72 h, the 24 well plates were centrifuged at 300 × *g* for 5 min. The supernatants were transferred to new tubes and stored at −80 °C until ready for use. Cytokine content of the supernatants was assayed using the ProcartaPlex Multiplex Immunoassay (ThermoFisher, EPXR360–26092–901) as directed by the manufacturer’s user guide. We used undiluted supernatant and probed for target molecules, including: GM-CSF, IFN-γ, IL-1β, IL-12p70, IL-13, IL-18, IL-2, IL-4, IL-5, IL-6, TNF-α, ENA-78 (CXCL5), G-CSF (CSF-3), IFN-α, IL-1α, IL-15, IL-28, IL-3, IL-31, LIF, M-CSF, IL-10, IL-17A, IL-22, IL-23, IL-27, IL-9, Eotaxin (CCL11), GRO-α (CXCL1), IP-10 (CXCL10), MCP-1 (CCL2), MCP-3 (CCL7), MIP-1α (CCL3), MIP-1β (CCL4), MIP-2α (CXCL2), and RANTES (CCL5). Supernatants representing one animal in the group boosted with KFE8-Ag85B-pulsed DCs are not included in the dataset due to a specimen collection error.

### RNA sequencing

BMDCs were cultured from bone marrow of C57BL/6 mice using rIL-4 and rGM-CSF. After 7 days of culture, BMDCs were harvested and plated in 24 well plates at a density of 5 × 10^5^/mL in 500 μL and treated with 10 ng/mL of bare KFE8 nanofibers. Following 4 or 16 h of incubation in a humidified incubator at 37 °C and 5% CO_2_, the cells were collected in TRIzol and RNA was isolated according to the manufacturer’s instructions. RNA sequencing and differential expression analysis were performed at Novogene Corporation, which includes read counts normalization, model dependent *p*-value estimation, and FDR value estimation based on multiple hypothesis testing. This is preceded by raw reads filtering, mapping clean reads to a reference genome using HISAT2, and determining FPKM values for all samples^76^. Differentially expressed genes were evaluated based on their log2 fold change and adjusted p-values. Those with |log2(fold change)| > 0 and adjusted *p*-values < 0.05 were considered to be differentially expressed and significant. Pathway analysis of significantly differentially expressed genes identified from RNA sequencing results was performed using the ReactomePA package^77^ (version 1.34.0) in R Studio v4.0.5. Pathways and biological processes with *p* value < 0.05 were considered significant. We used clusterProfiler^78^ (version 3.18.1) to compare the representation of enriched pathways between treatment groups, and the top upregulated and downregulated pathways at each time point are shown. Additionally, we utilized EnhancedVolcano^79^ to visualize the spread and magnitude of differentially expressed genes using a *p*-value cutoff of 0.01 and a log2 foldchange cutoff of 1.

### Statistical analysis

All data were analyzed using GraphPad Prism version 9 and presented as mean ± SEM). Significant differences were determined using a one-way ANOVA followed by an appropriate ad hoc test for differences due to treatment, or between treatment groups, as indicated in each figure legend. Statistical analysis of data sets containing only two experimental groups was conducted using a two-tailed unpaired *T* test. *P*-values of <0.05 were considered significant.

### Reporting summary

Further information on research design is available in the Nature Research Reporting Summary linked to this article.

## Supplementary information

Supplemental Files

REPORTING SUMMARY

## Data Availability

The transcriptomic data discussed in this publication are accessible through Zenodo at 10.5281/zenodo.5888396^80^.
