# Supplementary material for: Self-adjuvanting nanovaccines boost lung-resident CD4+ T cell immune responses in BCG-primed mice
Source: NPJ Vaccines. 2022 Apr 26;7:48. doi: 10.1038/s41541-022-00466-0 (PMC9043212; doi:10.1038/s41541-022-00466-0)
Supplement: Supplementary file 1 — Supplemental Files [file 41541_2022_466_MOESM1_ESM.pdf]

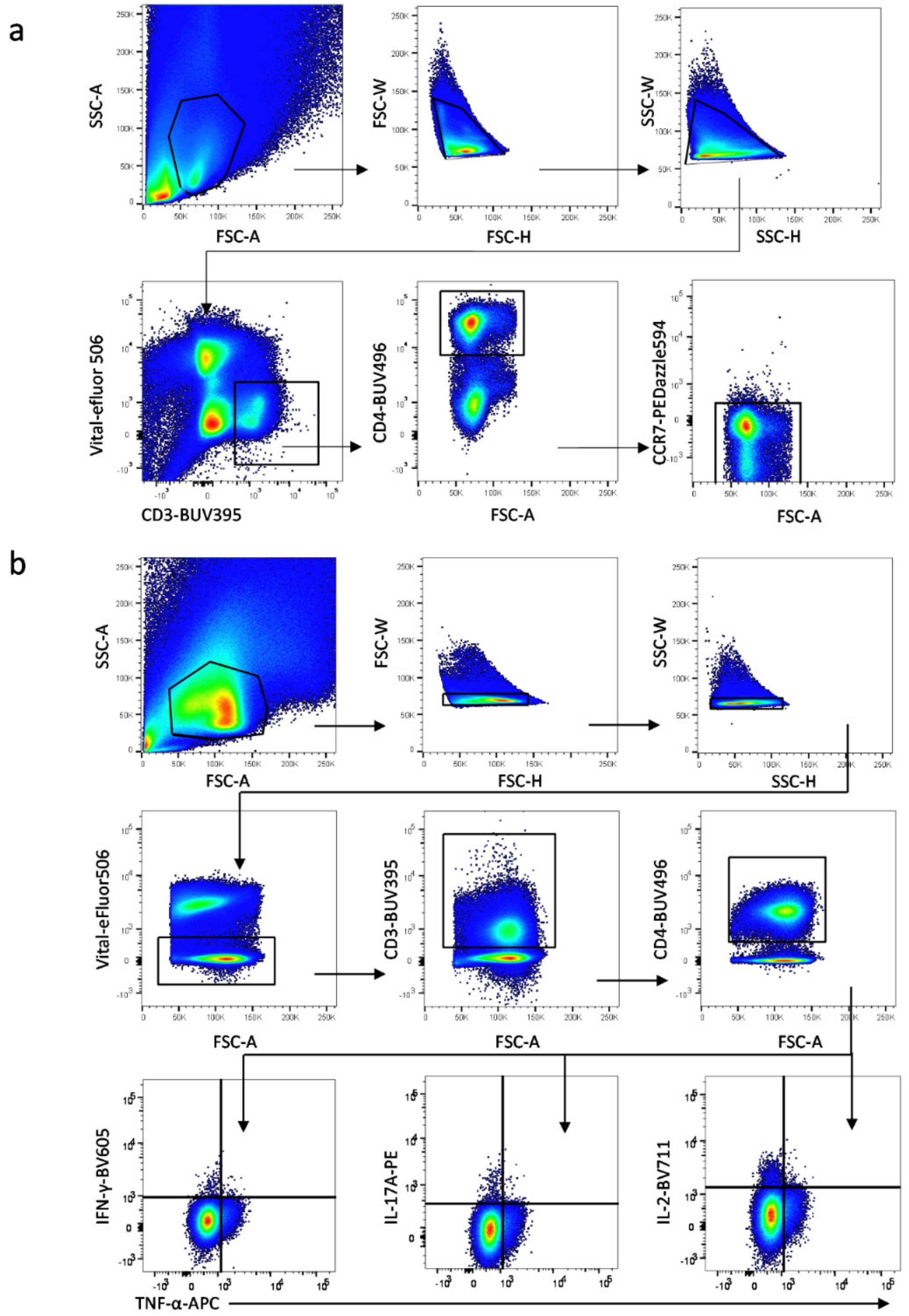

Supplemental Fig. 1 Gating Strategy for Flow Cytometric Analysis of Viable CD4+T cells. **a** Hierarchical selection of cells with Forward Scatter (FSC) and Side Scatter (SSC) characteristics of leukocytes, followed by selection of singlets through doublet exclusion, selection of viable cells based on exclusion of cells positively stained with a fixable viability dye, and selection of cells positive for CD3 and CD4 and negative for CCR7 for lung. **b** $CD3^+$ $CD4^+$ splenic lymphocytes were similarly selected following recall and further gated for cytokine expression (TNF-α, IFN-γ, IL-17A, IL-2). Polyfunctional activity was calculated through combinatorial Boolean analysis.

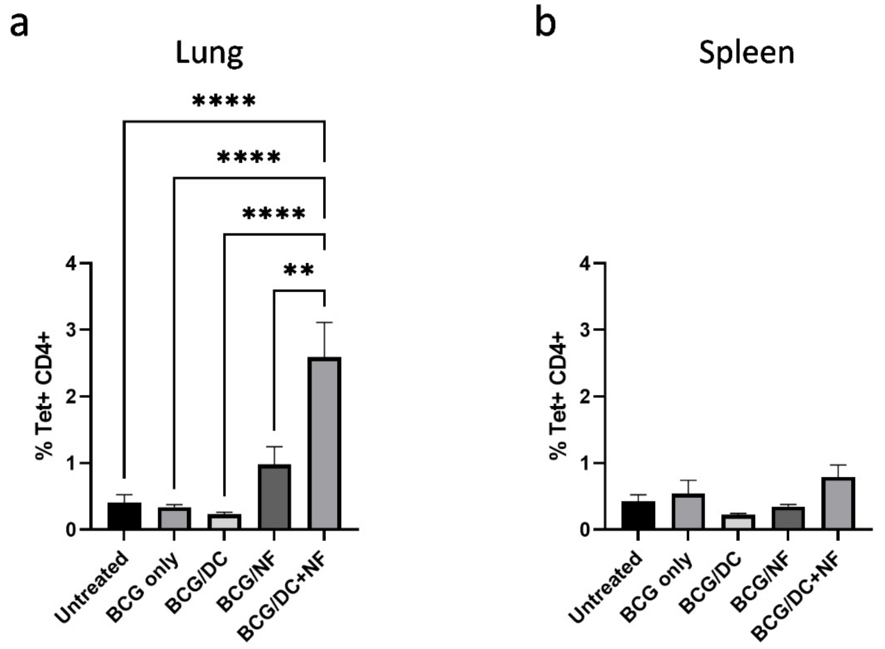

Supplemental Fig. 2 DC boosting of BCG-primed mice without KFE8-Ag85B does not increase tetramer+ CD4+ T cell populations. Tetramer^{+} CD4^{+} T cells were measured as in Figure 1 in **a** lung and **b** spleen. Data shown as mean ± SE. Significance was determined by one-way ANOVA comparing all groups followed by a Benjamini Krieger Yekutieli post hoc test for multiple comparisons to determine differences due to treatment and among treatment groups. *p < 0.05; **p < 0.01; ***p < 0.001; ****p< 0.0001.

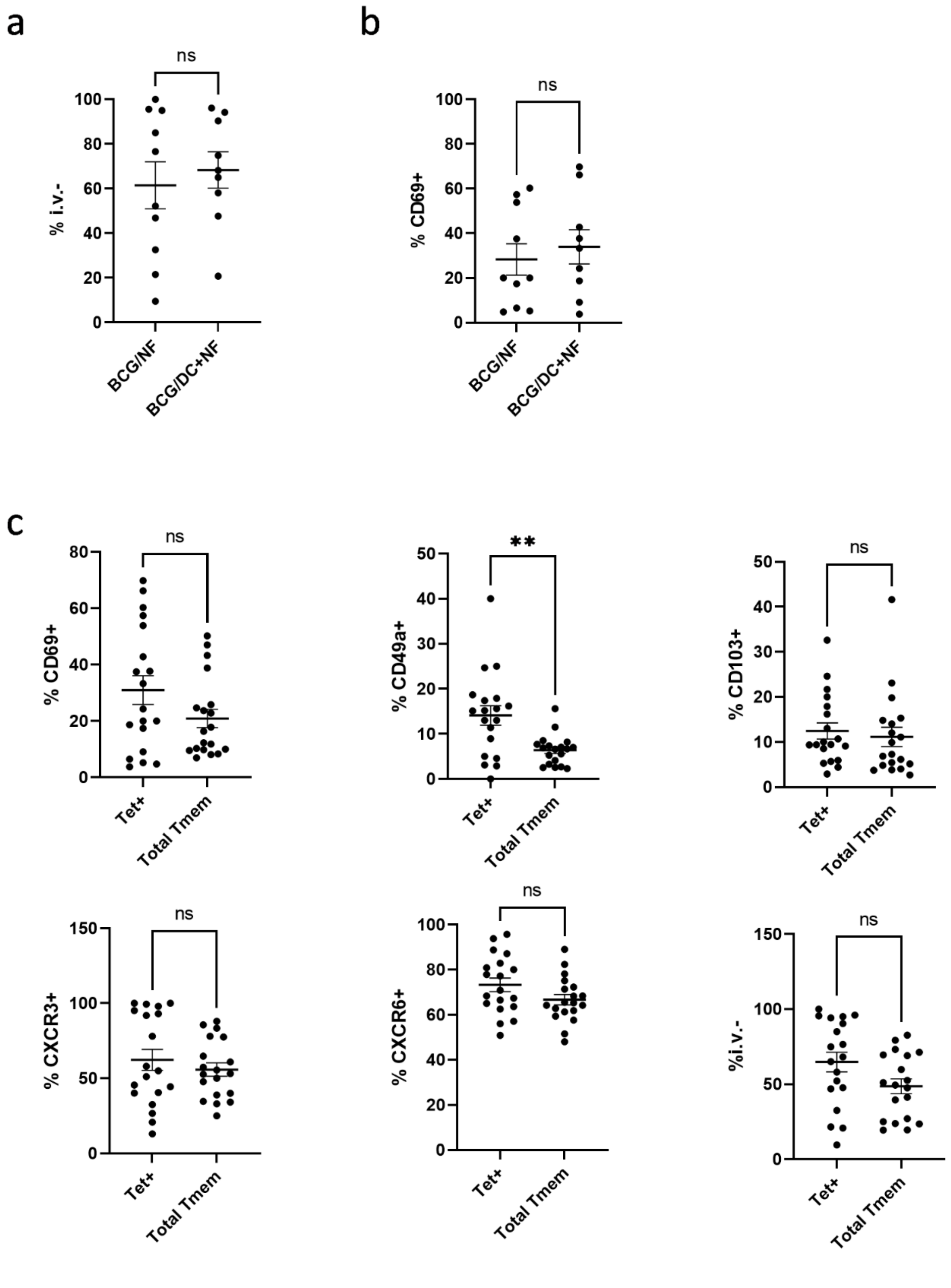

Supplemental Fig. 3 KFE8-Ag85B-induced memory T cell populations are similar to other effector memory and lung-resident memory T cells. Tetramer$^+$ memory CD4$^+$ T cells were analyzed for **a** i.v.- and **b** CD69$^+$ cells, most representative of tissue-residency, and compared between groups boosted with KFE8-Ag85B or DCs pulsed with KFE8-Ag85B. **c** Tetramer$^+$ cells from both boosted groups were combined and compared to the total memory CD4$^+$ T cells. Data shown as mean ± SE. Significance was determined by two-tailed unpaired T test. **$p < 0.01$.

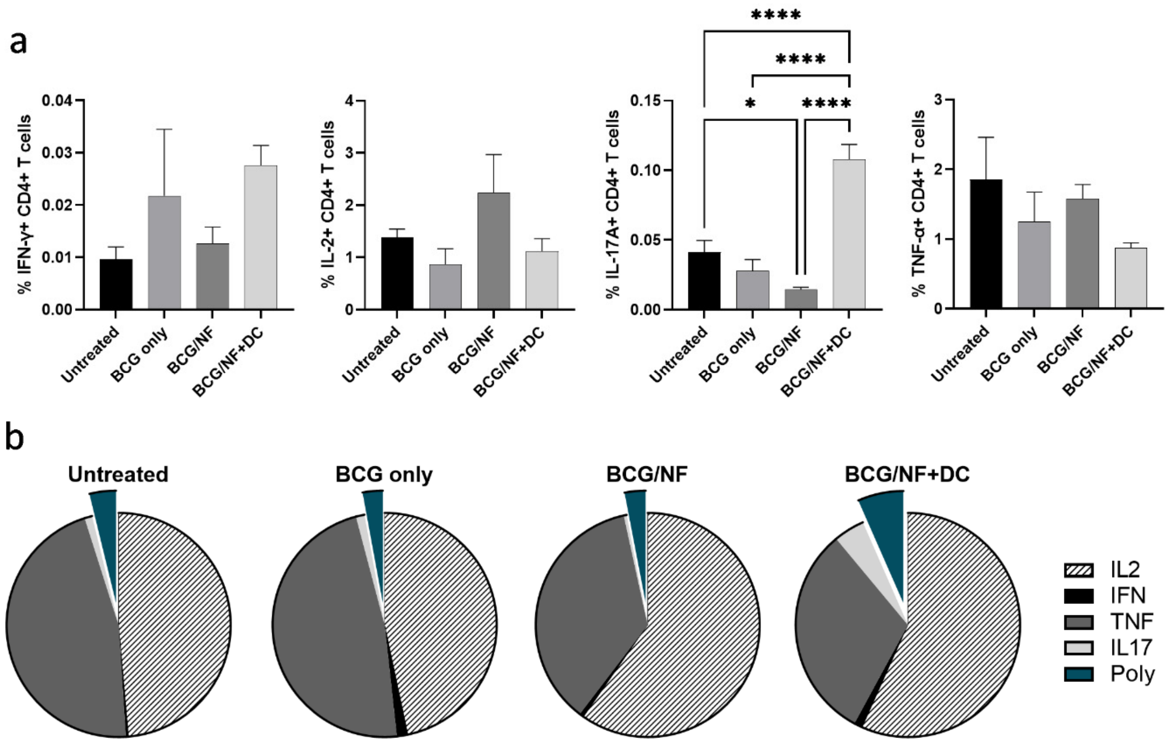

Supplemental Fig. 4 BCG-primed and KFE8-Ag85B boosted mice exhibit augmented cytokine production by splenocytes. Following antigen recall, cells were isolated from tissue culture plates and $CD3^+$ $CD4^+$ T cells were characterized for cytokine expression, including IFN-γ, TNF-α, IL-2, and IL-17A using surface and intracellular cytokine staining for flow cytometry. **a** $CD4^+$ T cells producing only one of these four cytokines are enumerated in panel. Data shown as mean ± SE. Pie charts in panel **b** represent all cytokine-producing $CD4^+$ T cells and indicate those which only produce one cytokine and those that produce two or more as polyfunctional. Significance was determined by one-way ANOVA followed by a Benjamini Krieger Yekutieli post hoc test for multiple comparisons to determine differences due to treatment and among treatment groups. $*p < 0.05$; $**p < 0.01$; $***p < 0.001$; $****p < 0.0001$.

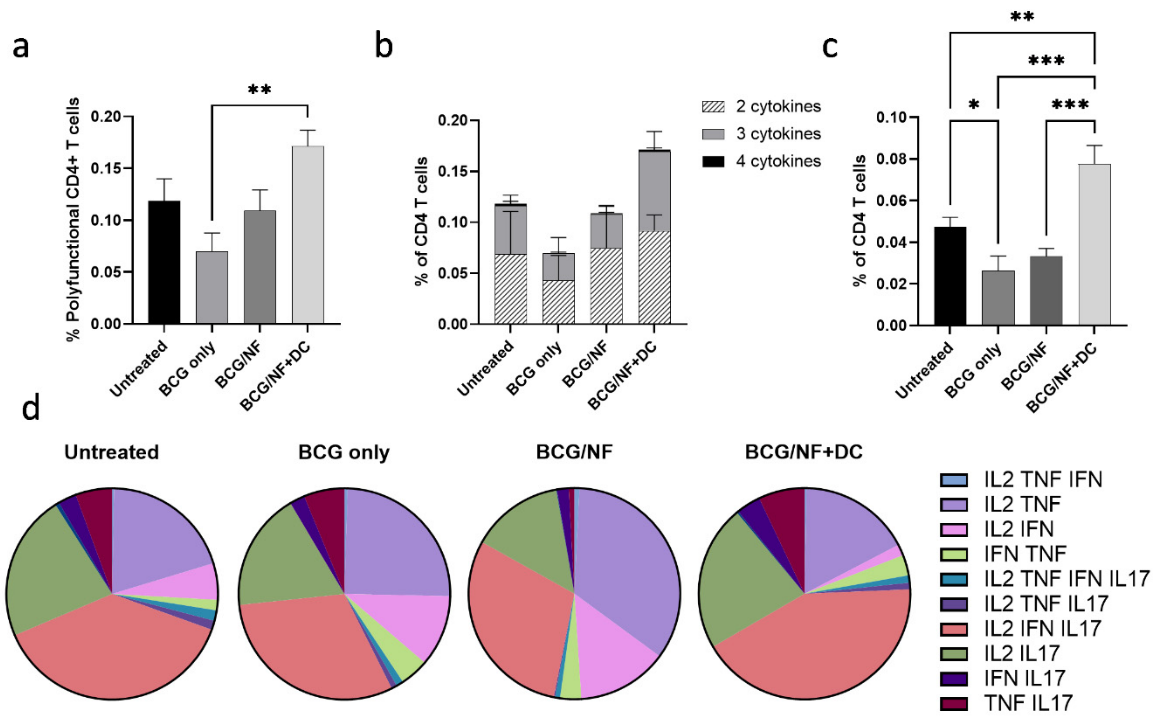

Supplemental Fig. 5 Boosting with KFE8-Ag85B-pulsed DCs significantly increased cytokine polyfunctionality of splenic CD4+ T cells compared with BCG prime alone. a $CD4^+$ T cells that two or more cytokines of interest were identified as polyfunctional. **b** Polyfunctional $CD4^+$ T cells were further split into three groups to quantify the percentage of $CD4^+$ T cells that produce 2 cytokines, 3 cytokines, or 4 cytokines. **c** $CD4^+$ T cells that express three cytokines are shown here to highlight significant differences. Data shown as mean ± SE. Significance was determined by one-way ANOVA followed by a Benjamini Krieger Yekutieli post hoc test for multiple comparisons to determine differences due to treatment and among treatment groups. *p < 0.05; **p < 0.01; ***p < 0.001; ****p< 0.0001. **d** All permutations of polyfunctionality were plotted in pie charts to visually compare frequency of unique populations of $CD4^+$ T cells among different treatment groups.
