# Supplementary material for: Self-adjuvanting nanovaccines boost lung-resident CD4+ T cell immune responses in BCG-primed mice
Source: NPJ Vaccines. 2022 Apr 26;7:48. doi: 10.1038/s41541-022-00466-0 (PMC9043212; doi:10.1038/s41541-022-00466-0)
Supplement: Supplementary file 2 — REPORTING SUMMARY [file 41541_2022_466_MOESM2_ESM.pdf]

nature portfolio

Corresponding author(s): Jai S. Rudra, Janice J. Endsley

Last updated by author(s): January 22, 2022

Reporting Summary

Nature Portfolio wishes to improve the reproducibility of the work that we publish. This form provides structure for consistency and transparency in reporting. For further information on Nature Portfolio policies, see our Editorial Policies and the Editorial Policy Checklist.

Statistics

For all statistical analyses, confirm that the following items are present in the figure legend, table legend, main text, or Methods section.

n/a	Confirmed	
☐	☒	The exact sample size (*n*) for each experimental group/condition, given as a discrete number and unit of measurement
☐	☒	A statement on whether measurements were taken from distinct samples or whether the same sample was measured repeatedly
☐	☒	The statistical test(s) used AND whether they are one- or two-sided<br>*Only common tests should be described solely by name; describe more complex techniques in the Methods section.*
☒	☐	A description of all covariates tested
☐	☒	A description of any assumptions or corrections, such as tests of normality and adjustment for multiple comparisons
☐	☒	A full description of the statistical parameters including central tendency (e.g. means) or other basic estimates (e.g. regression coefficient) AND variation (e.g. standard deviation) or associated estimates of uncertainty (e.g. confidence intervals)
☐	☒	For null hypothesis testing, the test statistic (e.g. *F*, *t*, *r*) with confidence intervals, effect sizes, degrees of freedom and *P* value noted<br>*Give P values as exact values whenever suitable.*
☒	☐	For Bayesian analysis, information on the choice of priors and Markov chain Monte Carlo settings
☒	☐	For hierarchical and complex designs, identification of the appropriate level for tests and full reporting of outcomes
☒	☐	Estimates of effect sizes (e.g. Cohen's *d*, Pearson's *r*), indicating how they were calculated

Our web collection on statistics for biologists contains articles on many of the points above.

Software and code

Policy information about availability of computer code

Data collection	BD FACSDIVA v9
Data analysis	FlowJo v10, BD FACSDiva v9, ReactomePA package79 (version 1.34.0) in R Studio v4.0.5, GraphPad Prism version 9

For manuscripts utilizing custom algorithms or software that are central to the research but not yet described in published literature, software must be made available to editors and reviewers. We strongly encourage code deposition in a community repository (e.g. GitHub). See the Nature Portfolio guidelines for submitting code & software for further information.

Data

Policy information about availability of data

All manuscripts must include a data availability statement. This statement should provide the following information, where applicable:

- Accession codes, unique identifiers, or web links for publicly available datasets
- A description of any restrictions on data availability
- For clinical datasets or third party data, please ensure that the statement adheres to our policy

The transcriptomic data discussed in this publication are accessible through Zenodo at https://doi.org/10.5281/zenodo.588839682.

Field-specific reporting

Please select the one below that is the best fit for your research. If you are not sure, read the appropriate sections before making your selection.

☒ Life sciences ☐ Behavioural & social sciences ☐ Ecological, evolutionary & environmental sciences

For a reference copy of the document with all sections, see nature.com/documents/nr-reporting-summary-flat.pdf

Life sciences study design

All studies must disclose on these points even when the disclosure is negative.

Sample size	Experiments included 4-10 animals per treatment, based on power calculation guided by previous/preliminary results
Data exclusions	No experimental data was excluded. Missing data points did occur as a result of technical issues (e.g. flow cytometry probe clog) that resulted in sample loss
Replication	Animal experiments to assess Trm were conducted three times using two different peptide synthesis batches with similar results
Randomization	Animals were from a single cohort purchased from a commercial vendor and randomly assigned to treatment
Blinding	Investigators were not blinded during collection or analysis due to the nature of the treatments including requirement for knowledge of priming dose to ensure proper boost.

Reporting for specific materials, systems and methods

We require information from authors about some types of materials, experimental systems and methods used in many studies. Here, indicate whether each material, system or method listed is relevant to your study. If you are not sure if a list item applies to your research, read the appropriate section before selecting a response.

Materials & experimental systems

n/a	Involved in the study	
☐	☒	Antibodies
☒	☐	Eukaryotic cell lines
☒	☐	Palaeontology and archaeology
☐	☒	Animals and other organisms
☒	☐	Human research participants
☒	☐	Clinical data
☒	☐	Dual use research of concern

Methods

n/a	Involved in the study	
☒	☐	ChIP-seq
☐	☒	Flow cytometry
☒	☐	MRI-based neuroimaging

Antibodies

Antibodies used

BV711 anti-CD45 (BD Biosciences, 563709)
Fixable Viability Dye eFluor 506 (eBioscience, 65-0866-14)
CD3 (BUV395, BD Biosciences, 563565)
CD4 (BUV496, BD Biosciences, 612952)
CCR7 (PE-Dazzle 594, Biolegend, 120122)
CD44 (PE-Vio770, Miltenyi Biotec, 130-102-377)
CD62L (Brilliant Violet 786, BD Biosciences, 564109)
CD69 (APC, Biolegend, 104514)
CD49a (VioBright FITC, Miltenyi Biotec, 130-107-592)
CD103 (Brilliant Violet 605, BD Biosciences, 748257)
CD127 (Brilliant Violet 650, BioLegend, 135043)
CXCR3 (PerCP-Cy5.5, BioLegend, 126514)
CXCR6 (PE, BioLegend, 151104)
IFN-γ (Brilliant Violet 605, Biolegend, 505840)
TNF-α (APC, Biolgened, 506108))
IL-2 (Brilliant Violet 711, Biolegend, 503837)
IL-17A (PE, Biolegend, 506904)
MHC-II Ag85B tetramer (I-A(b) FQDAYNAAGGHNAVF), NIH Tetramer Core Facility

Validation

All antibodies were purchased directly from reputable vendors (listed above) and came with quality certificates of validation. Additional validation was performed using ultracomp beads to assess quality and performance

Animals and other organisms

Policy information about studies involving animals; ARRIVE guidelines recommended for reporting animal research

Laboratory animals	Female C57BL/6 mice supplied by Jackson Labs, 6-8 weeks old
Wild animals	study did not involve wild animals
Field-collected samples	study did not utilize field collected samples
Ethics oversight	University of Texas Medical Branch Institutional Animal Care and Use Committee

Note that full information on the approval of the study protocol must also be provided in the manuscript.

Flow Cytometry

Plots

Confirm that:

☒ The axis labels state the marker and fluorochrome used (e.g. CD4-FITC).

☒ The axis scales are clearly visible. Include numbers along axes only for bottom left plot of group (a 'group' is an analysis of identical markers).

☒ All plots are contour plots with outliers or pseudocolor plots.

☒ A numerical value for number of cells or percentage (with statistics) is provided.

Methodology

Sample preparation	Lung and spleen cells isolated through disruption including use of DNASE and collagenase in processing (full details are included in manuscript methods).
Instrument	Becton Dickinson LSR II, Fortessa
Software	BD FACSDiva and FlowJo v10 software
Cell population abundance	Sorting experiments were not performed. For analytical flow cytometry, all events in isolated tissue were collected which averaged 10 million in lung and 2 million in spleen which is more enriched in lymphocytes versus lung.
Gating strategy	Gating was performed by selection of FSC/SSC characteristics of live leukocytes, selection of singlets, selection of viable cells excluding a viability dye, followed by additional downstream gating to select CD3+CD4+ populations. Analysis of activation, memory, or tissue resident phenotype was subsequently determined from viable CD3+CD4+ singlets.

☒ Tick this box to confirm that a figure exemplifying the gating strategy is provided in the Supplementary Information.
